# Novel killer yeasts and toxins from the gardens of fungus-growing ants

**DOI:** 10.1128/aem.02246-25

**Published:** 2026-01-21

**Authors:** Rodolfo Bizarria, Jack W. Creagh, Renato A. Corrêa dos Santos, Lily L. Givens, Sara A. Coss, Tanner J. Badigian, Adrian V. Chavez, Rim T. Tekle, Noah Fredstrom, F. Marty Ytreberg, Maitreya J. Dunham, Andre Rodrigues, Paul A. Rowley

**Affiliations:** 1 Department of Biological Sciences, University of Idaho, Moscow, Idaho, USA; 2 Department of General and Applied Biology, São Paulo State University (UNESP), Institute of Biosciences, Rio Claro, São Paulo, Brazil; 3 Laboratory of Computational, Evolutionary, and Systems Biology, Center for Nuclear Energy in Agriculture, University of São Paulo, Piracicaba, São Paulo, Brazil; 4 Department of Genome Sciences, University of Washington, Seattle, Washington, USA; 5 Department of Physics, University of Idaho, Moscow, Idaho, USA; 6 Institute for Modeling Collaboration and Innovation, University of Idaho, Moscow, Idaho, USA

**Keywords:** killer yeasts, killer toxins, antifungals, budding yeast, fungus-farming ants

## Abstract

Killer toxins are proteinaceous antifungal molecules produced by yeasts, with activity against a wide range of human and plant pathogenic fungi. Fungus gardens of attine ants in Brazil were surveyed to determine the presence of killer toxin-producing yeasts and to define their antifungal activities and ecological importance. Our results indicate that 10 out of 59 yeast species isolated from fungal gardens are killer yeasts. Killer yeasts were less likely to inhibit the growth of yeasts isolated from the same environment but more effective at inhibiting yeasts isolated from other environments, supporting a role for killer yeasts in shaping the community composition. All killer yeasts had genome-encoded killer toxins and lacked cytoplasmic toxin-encoding elements (i.e., double-stranded RNA satellites and linear double-stranded DNAs). Of all the killer yeasts associated with attine ants, *Candida sinolaborantium* (strain LESF 1467) showed a broad spectrum of antifungal activities against 39 out of 69 (57%) of yeast strains tested for toxin susceptibility. The complete genome sequence of *C. sinolaborantium* LESF 1467 identified a new killer toxin, Ksino, with similarities in the primary sequence and tertiary structure to the *Saccharomyces cerevisiae* killer toxin named Klus. Surveys of publicly available genome databases identified homologs of Ksino in the genomes of yeast strains of *Saccharomycetes* and *Pichiomycetes*, as well as other species of Ascomycota and Basidiomycota filamentous fungi. This demonstrates that killer yeasts can be widespread in attine ant fungus gardens, possibly influencing the fungal community composition and the importance of these complex microbial communities in the discovery of novel antifungal molecules.

Killer yeasts were first described in *Saccharomyces cerevisiae* in the 1960s, after which many strains and species of yeasts and yeast-like fungi were also observed to secrete antifungal killer toxins (1–6). Killer toxins can have a range of antifungal activities from narrow to broad specificities, affecting cells from close or distantly related fungal species (7–9). The prevalence of killer yeast strains in natural environments is reported between 5% and 33% for positive killer phenotypes (10–17) but can also be as high as 59% in yeast associated with wine production (18). Killer toxins are known to be active against human and plant pathogenic yeasts (19–23) and can control spoilage organisms that are important for agriculture and the food industry (24, 25). The rise in acquired drug resistance and the emergence of drug-resistant fungal pathogens justify the search for novel antifungal killer toxins from diverse natural environments, including insect-associated fungal communities.

Killer yeast communities have been previously reported in fungus-growing ant colonies (Hymenoptera: tribe Attini: subtribe Attina, hereafter called “attine ants”). Attine ants established a long-term obligate mutualism with basidiomycete fungi in the *Agaricaceae* and *Pterulaceae* families, using them as the primary food source for their colonies (26–30). According to their cultivars and foraging substrates, each species approaches fungiculture differently, and these interactions are considered a model for studying the evolution of mutualisms (28, 31). Moreover, attine ants are key ecological agents in the Neotropics as dominant herbivores, promoting nutrient cycling, soil fertility, and seed dispersal (32–36). The foraging behavior of attine ants also contributes to the presence of a large and complex community of environmental yeasts in fungus gardens (37–41). Fungus gardens are likely an important source of new killer toxins due to the high concentration of simple sugars (42, 43), large density of yeasts and filamentous fungi, and the hypothesized role in garden “immunity” for yeasts—all key elements for species competition (37–39, 44–46). Killer yeasts are primarily associated with the basidiomycete fungus farmed by attine ants, the bodies of ants, and the leaves foraged by ants (41, 47, 48). In prior studies, killer positive phenotypes have been identified in 5% to 11% of the interactions between yeasts derived from attine ant fungus gardens (41, 47).

Killer toxins can modify the community composition of yeasts as an outcome of competition or cooperation between killer yeasts, killer toxin-susceptible yeasts, and killer toxin-resistant yeasts that are determined by spatial distribution, pH, and ploidy (49–51). Killer toxin production also plays a potential role in yeast dispersal, resource consumption among species, and invade or prevent invasion from competitors (7, 52–55). The coexistence of the different phenotypes is known to be affected by environmental parameters, including nutrient availability (50, 53), cell density (56, 57), pH, and temperature (49, 58, 59), which can reflect the production and distribution of killer toxins in spatially structured environments (7, 50, 60–62). On the other hand, killer toxins are often reported to play a role in interference competition in environments that enforce the interaction between toxin-producing and neighboring susceptible cells (such as well - mixed habitats like laboratory co-cultures and wine fermentations), changing the community structure and function, as an outcome of competition or cooperation between killer yeasts, killer toxin-susceptible yeasts, and killer toxin-resistant yeasts (7, 16, 18, 63).

Killer toxins are encoded by different genetic elements, including chromosomal genes, extrachromosomal elements such as non-autonomous double-stranded RNA (dsRNA) satellites that are maintained by mycoviruses, and autonomous linear double-stranded DNAs (dsDNA) (59, 64–66). dsRNA satellites have been previously found to be associated with *Hanseniaspora uvarum, Pichia kluyveri, Saccharomyces* spp., *Torulaspora delbrueckii*, and *Zygosaccharomyces bailii* (67–73), while dsDNA plasmids have been identified in the genera *Babjevia, Debaryomyces, Kluyveromyces, Millerozyma*, and *Pichia* (74–76). The association of the killer phenotype with the presence of dsRNA satellites has been most thoroughly studied in *Saccharomyces cerevisiae* (77, 78). For example, in a survey of 1,270 *S. cerevisiae* isolates, the frequency of killer yeasts was 50%, and 60% of these killer yeasts had dsRNA satellites (77). Killer toxin production by the remaining 40% of killer yeasts was closely correlated with the chromosomal killer toxin gene *KHS1*. Given the many killer yeasts described, only a few studies have identified chromosomal genes encoding the toxins responsible for the observed antifungal effects. Chromosomally encoded killer toxins and their homologs, including KKT (K1-like killer toxins), KHR, KHS, SMKT, KP4-like, PMKT, and other *Pichia* toxins, have been described in many different species of fungi (79–85). Despite the prevalence of genomic-encoded toxins, some dsRNA satellite-encoded toxins were observed to have genomic homologs in different Saccharomycotina lineages (83, 86, 87). However, the frequency of killer yeasts and the prevalence of dsRNA satellites and dsDNA extrachromosomal elements in different lineages of Saccharomycotina and yeast-like fungi are still understudied.

In this study, we explore the nature and prevalence of the killer phenotype in different Saccharomycotina lineages associated with attine ant fungus gardens, which are habitats that are rich in yeasts, shedding light on their putative ecological role in this environment. It was revealed that fungus gardens harbor diverse killer yeasts with narrow and broad antifungal activities. Unlike the well-studied killer yeasts of the *Pichia* and the *Saccharomyces* genera, attine ant-associated killer yeasts were devoid of cytoplasmic toxin-encoding elements such as linear dsDNA plasmids and dsRNA satellites. Therefore, to identify the gene(s) responsible for killer toxin production, a genome mining approach was used by determining the whole-genome sequence of the killer yeast *Candida sinolaborantium* that displayed a broad spectrum of antifungal activities against many species of yeasts, including human pathogens. This approach identified a new killer toxin named Ksino. This novel killer toxin has homologs in both yeasts and filamentous fungi and shares a similar predicted structure to an *S. cerevisiae* killer toxin named Klus. Collectively, it was found that fungus gardens of attine ants can be used to explore the ecological role of killer yeasts and discover new killer toxins for possible future application as natural antifungals.

## RESULTS

### Fungus gardens of attine ants harbor killer yeasts

**FIG 1:**
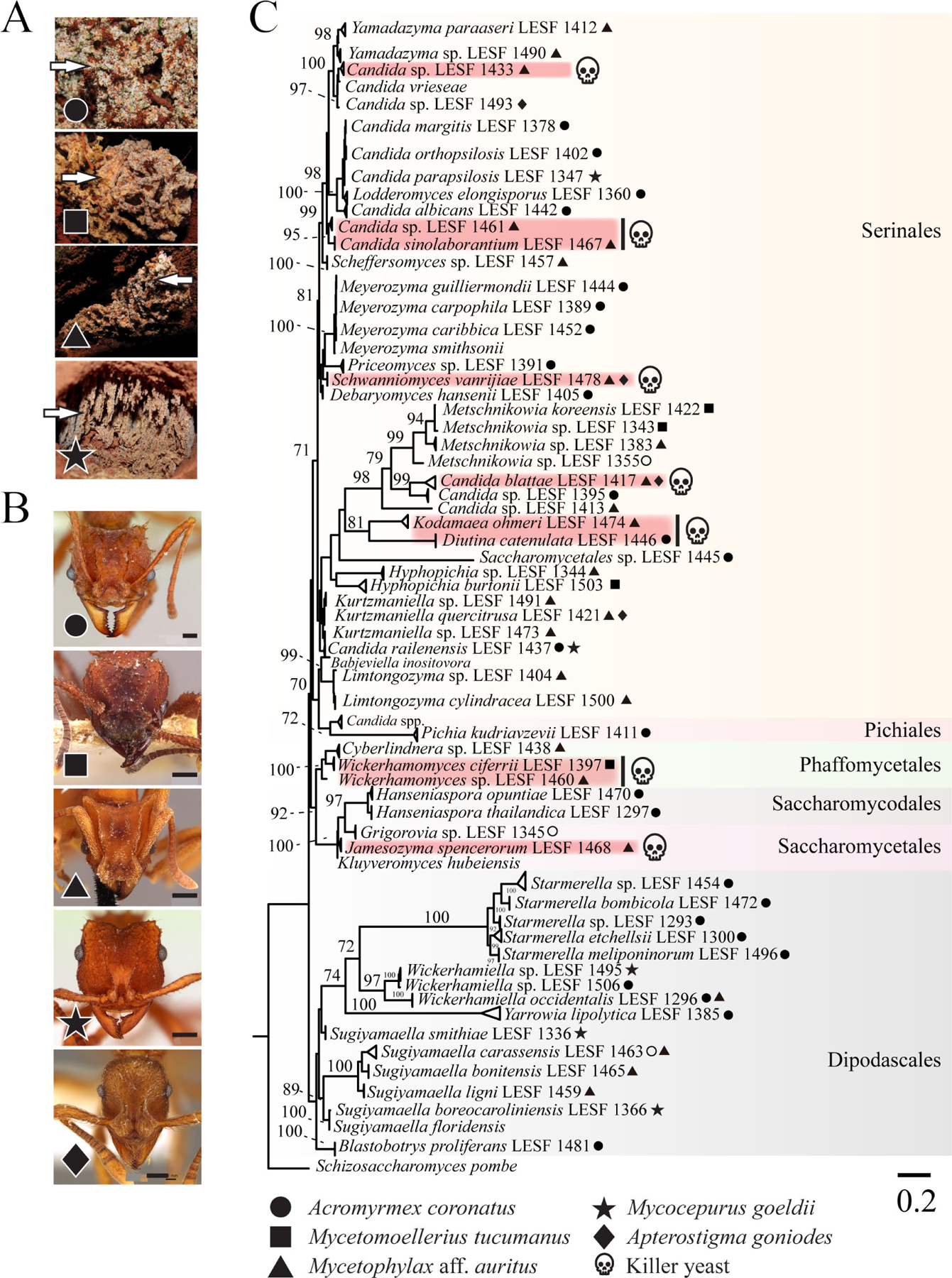
Fungus gardens of different attine ants harbor different killer yeasts. (A) Images of fungus gardens of different ant species from which yeasts were isolated (arrowheads). The gardens of the leaf-cutting ant *Acromyrmex coronatus* (circle), the non-leaf-cutting ant *Mycetomoellerius tucumanus* (square), and the lower attines *Mycetophylax aff. auritus* (triangle) and *Mycocepurus goeldii* (star). The fungus garden of *Apterostigma goniodes* is not represented. (B) Representative images of attine ant species: *Acromyrmex coronatus* (CASENT0173791, April Nobile) (circle); *Mycetomoellerius tucumanus* (CASENT0909391, Will Ericson) (square); *Mycetophylax auritus* (CASENT0901666, Ryan Perry) (triangle); *Mycocepurus goeldii* (CASENT0173988, April Nobile) (star); and *Apterostigma goniodes* (CASENT0922040, Michele Esposito) (diamond). The scale bar on all images represents 0.2 mm. Photos of ants were downloaded from www.antweb.org. (C) Phylogenetic relationship of yeasts isolated from fungal gardens as inferred by maximum likelihood (ML) and the rRNA gene. Symbols in tree leaves indicate the isolation source of yeasts, as depicted in panels A and B. Red shading and skulls denote the species confirmed as killer yeasts. The phylogenetic clades were highlighted by order. *Schizosaccharomyces pombe* was positioned as an outgroup. The numbers on the branches are ultrafast bootstrap values (values higher than 70 are shown), and the scale bar denotes the number of nucleotide substitutions per site.

To assess the diversity of yeasts associated with fungus-growing ants and their potential for antifungal toxin production, fungus gardens of four ant fungiculture systems were surveyed from 28 nests in four Brazilian cities (Fig. 1A; Table S1). The ant species included the leaf-cutting ant *Acromyrmex coronatus*, the non-leaf-cutting ant *Mycetomoellerius tucumanus*, and the lower attines *Mycetophylax* aff. *auritus* and *Mycocepurus goeldii* that cultivate Agaricaceae fungi and *Apterostigma goniodes* that cultivates Pterulaceae fungi (Fig. 1B). Ants were selected to broadly represent different fungiculture systems, foraging, and preparation behaviors and the yeast microbiota associated with fungus gardens. Yeasts were isolated from fungus gardens by culturing on synthetic growth media. Microsatellite amplification, sequencing of the D1/D2 domain of the large subunit ribosomal RNA gene, and phylogenetic reconstruction were used for taxonomic analysis (Fig. 1A; Table S2). The 180 isolated yeast strains comprised 59 species belonging to eight families and six orders from the Saccharomycotina subphylum previously characterized in a large study of yeasts and yeast-like fungi associated with fungus gardens of attine ants (Fig. 1C; Table S1) (41). Of the yeasts isolated, 91 were associated with *Acromyrmex coronatus*, 13 with *Mycetomoellerius tucumanus*, 42 with *Mycetophylax* aff. *auritus*, 25 with *Mycocepurus goeldii*, and 9 with *Apterostigma goniodes* (Table S1) (41).

**FIG 2:**
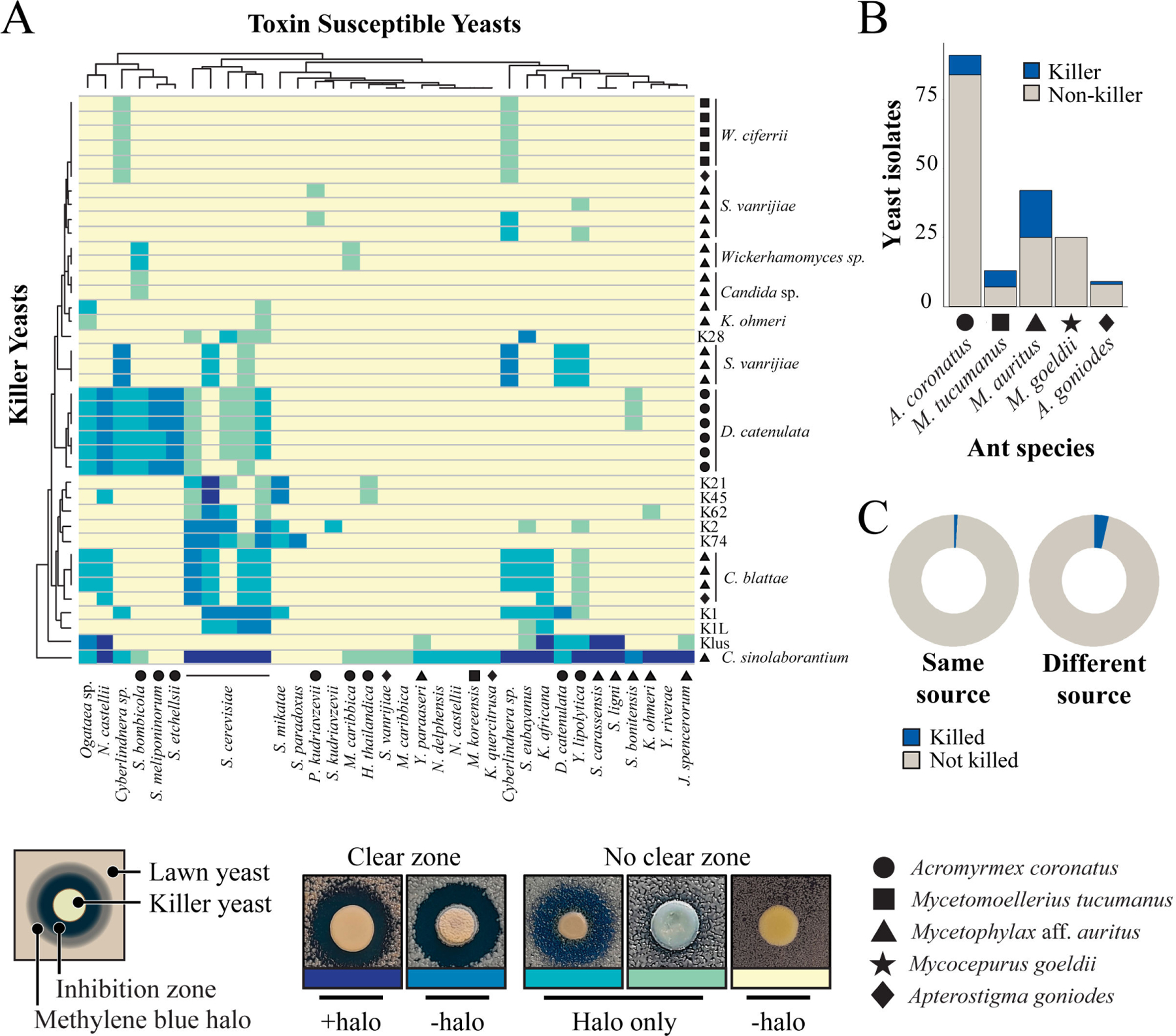
Killer yeasts from fungus gardens have unique antifungal activities. (A) Heatmap with the interactions between canonical *Saccharomyces* killer yeasts (K1, K1L, K2, Klus, K21, K28, K45, K62, and K74), killer yeasts associated with ants, and killer toxin susceptible strains. Origins of the ant-associated killer yeasts; *Acromyrmex coronatus* (circle); *Mycetomoellerius tucumanus* (square); *Mycetophylax aff. auritus* (triangle); *Apterostigma goniodes* (diamond). Killer toxin activity was qualitatively assessed based on the presence and size of growth inhibition zones and/or methylene blue staining around killer yeasts (representative images of these phenotypes are shown). Darker colors on the heatmap represent a more prominent killer phenotype, with yellow indicating no observable killer phenotype. Clusters on the dendrograms connecting individual killers or susceptible yeasts indicate similar susceptibilities to killer toxins or antifungal activities. (B) Number of killer yeasts and non-killers associated with the different attine ant fungicultures. (C) Number of killer toxin-positive and -negative interactions between yeasts from attine ant environment and susceptible strains (Table S1 and File S1), and growth inhibition by killer yeasts is different among the same or different fungicultures and locations.

From the 59 yeast species identified, ten species had isolates that were able to produce killer toxins (10 out of 59; 17% killer yeasts) (Fig. 1C). Killer toxin production was scored by the absence of growth around a killer yeast or the staining of the lawn yeast with the redox indicator methylene blue (as an indicator of cell death) at pH 4.6. This pH was used as many previously described killer toxins are active in these conditions, and it is close to the pH observed in fungus gardens where these yeasts were isolated (pH 5.1–5.4). The identified killer yeasts were effective at inhibiting strains that were either previously determined to be susceptible to killer toxins (83), human pathogenic yeasts, or yeasts randomly selected from fungus gardens and other sources (Fig. 2A; Fig. S1 and File S1). From the attine ant gardens, *Candida blattae*, *Candida sinolaborantium*, *Candida* sp. (closest to *Candida temnochilae*), *Candida* sp. (closest to *Candida membranifaciens*), *Diutina catenulata, Jamesozyma spencerorum, Kodamaea ohmeri, Schwanniomyces vanrijiae, Wickerhamomyces ciferrii*, and *Wickerhamomyces* sp. (closest to *Wickerhamomyces rabaulensis*) were identified as killer yeasts. These yeasts had unique antifungal activities when compared to other previously identified *Saccharomyces* killer toxins (K1, K1L, K2, K21, K28, K45, K62, K74, and Klus), and such diversity of activities has been observed in a previous screen for *Saccharomyces* killer yeasts (77). Killer yeasts were only absent from the fungus gardens of *M. goeldii*, but were recovered from the fungus gardens of *Acromyrmex coronatus* (7% killer yeasts; 6 out of 91 isolates), *Mycetomoellerius tucumanus* (46%; 6 out of 13), *Mycetophylax* aff. *auritus* (43%, 18 out of 42); and *Apterostigma goniodes* (22%; 2 out of 9) (Fig. 2B). A total of 32 out of 180 (18%) yeasts from attine ants were also capable of inhibiting the growth of susceptible yeasts, including human pathogenic yeasts such as *Candida albicans, Candida glabrata*, and *Candida auris* (Fig. S1). The attine ant-associated killer yeasts *J. spencerorum* LESF 1468 and *S. vanrijiae* LESF 1521 could only inhibit the growth of pathogenic yeasts (Fig. S1 and File S1). Many killer yeasts from the same species had similar antifungal activities, as demonstrated by their phenotypic clustering (Fig. 2A). Specifically, *Candida blattae* killer yeasts had similar antifungal activities even when isolated from the fungicultures of different ant species (compare *Candida blattae* isolates from *Mycetophylax* aff. *auritus* and *Apterostigma goniodes)* (Fig. 2A). However, there were isolates of *S. vanrijiae* killer yeasts from the fungiculture of *Mycetophylax* aff. *auritus* with strikingly different spectra of antifungal activities, which suggests that isolates of the same species were not always clonal. *Candida sinolaborantium* (strain LESF 1467) had the broadest range of antifungal activity, inhibiting 57% of the 69 strains challenged (Fig. 2A; Fig. S1 and File S1). Comparing interactions between ant-associated killer yeasts and susceptible lawn strains from fungus gardens and other environmental sources found that growth inhibition was more prevalent for yeasts from different sources (chi-squared test, *P*-value < 0.01) (Fig. 2C; Table S3). Specifically, only 1% (6 of 647) of interactions between ant-associated yeasts from the same type of fungiculture resulted in growth inhibition. In contrast, 4% (301 of 8,353) of interactions between ant-associated killer yeasts and yeasts from fungus gardens and other environmental sources isolation sources resulted in growth inhibition.

The ability to produce killer toxins is often associated with the presence of linear dsDNA plasmids or mycovirus-associated dsRNA satellites. We assayed all attine ant-associated killer yeasts for the presence of these genetic elements using solvent extraction of nucleic acids, cellulose chromatography, and gel electrophoresis (88). In particular, the use of cellulose chromatography selectively enriches dsRNAs derived from mycoviruses and satellites, reducing the false-negative rate compared to solvent extraction alone and without the bias of reverse-transcriptase PCR (77). However, we did not find that any of the isolated attine ant-associated strains possess dsRNAs (i.e., viruses and satellites) or dsDNA cytoplasmic elements (i.e., linear plasmids) (Fig. S2 and S3). dsRNA viruses have been found in many different species of yeasts without satellite dsRNAs (89–92); thus, we broadened the survey to include all attine-associated yeasts but again did not identify any dsRNA viruses. This suggested that the killer toxins associated with yeasts from fungus gardens are genome-encoded and that dsRNA viruses, dsRNA satellites, and DNA plasmids are rare or not associated with attine ant species in the geographical area sampled.

### Characterization of the killer yeast *Candida sinolaborantium*

**FIG 3:**
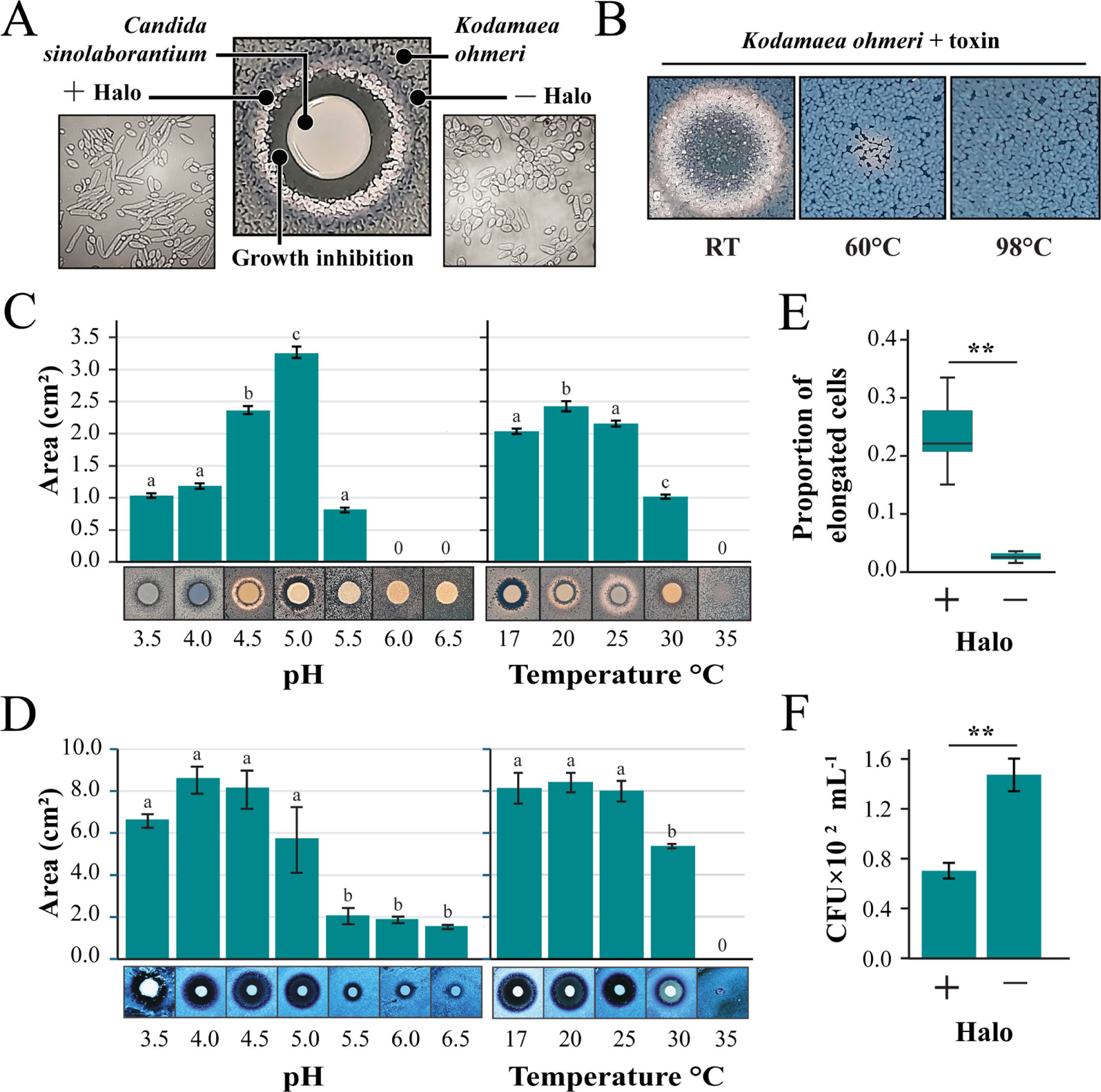
The antifungal activities of *Candida sinolaborantium*. (A) Growth phenotypes associated with the co-culture of the killer yeast *C. sinolaborantium* with *Kodamaea ohmeri* at RT and pH 4.6. Cell morphology of *K. ohmeri* inside (+Halo) and outside (−Halo) the white halo around *C. sinolaborantium* was evaluated by microscopy. (B) The antifungal activity of protein precipitates derived from the spent growth medium of *C. sinolaborantium* and the heat stability of these activities at 60°C and 98°C. Killer yeast activity of *C. sinolaborantium* under different pH and temperature conditions against (C) *K. ohmeri* and (D) *C. castellii*. For all pH and temperature data, the means were significantly heterogeneous (one-way ANOVA, *P* < 0.01). Means with the same letter are not significantly different from each other (Tukey–Kramer test, *P* > 0.05). (E) Proportion of elongated to normal cells of *K. ohmeri* inside (+Halo) and outside (−Halo) the white halo around *C. sinolaborantium*, as depicted in (A). (F) The same growth areas as shown in (E), but CFUs were measured to indicate *K. ohmeri* cell survival. Asterisks indicate different means for the Welch two-sample *t*-test (*P*-value < 0.01). All error bars are standard error (*n* > 3).

*C. sinolaborantium* was identified as a killer yeast with a broad antifungal spectrum that could inhibit 39 of the 69 yeast strains tested (57%) (Fig. 2A; Fig. S1). In contrast, the next most potent killer yeasts could only inhibit 11 of the strains tested (16%). *C. sinolaborantium* was also able to cause large zones of growth inhibition in many different species of yeasts that were resistant to other killer yeasts (Fig. 2A). After several days of incubation, we observed that *K. ohmeri* produced a striking white halo between a zone of growth inhibition and methylene blue staining when challenged by the killer yeast *C. sinolaborantium* (Fig. 3A). This phenotype was unique to the pairing of *C. sinolaborantium* and *K. ohmeri* and was distinct from the growth inhibition caused by the killer toxin K62 (the only other killer toxin capable of inhibiting *K. ohmeri*) (Fig. 2A). The white halo appeared to be the raised growth of *K. ohmeri* from the surface of the agar plate (Fig. S4). When cells in this area (+halo) were observed under a microscope, they appeared to be elongated compared to cells of *K. ohmeri* outside of the halo (−halo) (Fig. 3A). The killer toxin (or toxins) secreted by *C. sinolaborantium* was precipitated by ethanol and remained active when incubated at room temperature (RT) but was inactivated by heating to 60°C and 98°C (Fig. 3B). The inhibition of *K. ohmeri* by *C. sinolaborantium* was optimal at pH 5, with loss of killing at pH > 5.5 (Fig. 3C; Table S4). The optimal temperature for killing *K. ohmeri* by *C. sinolaborantium* was 20°C (Fig. 3C; Table S4). Using *Candida castellii* as a sensitive lawn indicated a broader optimum temperature (17°C–25°C) and pH (3.5–5.0) (Fig. 3D; Table S4 and S5). In both cases, there was no killer activity at 35°C (Fig. 3C and D; Table S5). Further analysis of the white halo of *K. ohmeri* (+halo) revealed an eightfold increase in cell elongation when compared to untreated cultures of *K. ohmeri* (−halo) (Fig. 3E; Table S6). Cells of *K. ohmeri* from the white halo or untreated culture were seeded on agar to measure the viability by colony-forming units (CFUs). Cells isolated from the white halos showed a 2.1-fold reduction in CFUs compared to *K. ohmeri* isolated outside of the halos (Fig. 3F; Table S7). Ultrafiltration of culture media was used to determine the approximate molecular weight of the antifungal molecules produced by *C. sinolaborantium* (Fig. S5). Antifungal activity was observed for fractions that enriched molecules >100 kDa and >30 kDa. Raw filtrates and concentrated ethanol-precipitated filtrates were used to challenge a panel of yeasts previously shown to be susceptible to toxins produced by *C. sinolaborantium* (Fig. 2). The spent culture medium showed less antifungal activity compared to the precipitated medium, with the latter capable of inhibiting 21/29 susceptible yeasts that were inhibited in killer assays on agar (Fig. S5). Antifungal activities of crude or precipitated growth medium were similar before and after passage through a filter with a molecular weight cut-off (MWCO) of 100 kDa and were mostly retained in the MWCO 30 kDa filter. Only weak antifungal activity was detectable for the fractions that passed through the MWCO 30 kDa filter (Fig. S5). The appearance of white halos was observed using fractions captured by filters with MWCO 100 kDa and MWCO 30 kDa when tested against *K. ohmeri*, but not with a MWCO 5 kDa. Together, these data show that *C. sinolaborantium* is a killer yeast with a broad spectrum of antifungal activities, with temperature and pH optima similar to those of other previously described yeast killer toxins, but with the ability to induce cell elongation in *K. ohmeri*. Fractions from ultrafiltration are consistent with the antifungal activities of *C. sinolaborantium*, suggesting either the production of multiple chromosomally encoded killer toxins with similar antifungal properties or perhaps large oligomers or complexes of a single >30 kDa killer toxin.

### Ksino: a novel killer toxin produced by *C. sinolaborantium* with structural homology to Klus

To determine the gene or genes responsible for killer toxin production by *C. sinolaborantium*, the genome sequence of the yeast was determined (Table S8). The resulting 11.21 Mb assembly resulted in a BUSCO completeness (complete and single-copy BUSCOs) of around 98% using the saccharomycetes_odb10 data set (93), with GC content of 49%, an N50 of 155,979 bp, and a total of 5,951 protein-coding genes (Table S8). To identify genome-encoded killer toxins in the *C. sinolaborantium* proteome, BLASTp was performed using a database of known yeast killer toxins (Table S9). This approach identified 12 toxin-like candidates, all with a sequence alignment length greater than 100 amino acids and an identity of at least 24%, compared to any known killer toxin (Table S10). One *C. sinolaborantium* protein had a 28% identity and 42% similarity over an alignment length of 151 amino acids with the *S. cerevisiae* killer toxin Klus and was named Ksino (Killer from sinolaborantium) (GenBank Accession: XAT76252) (Table S10) (94).

**FIG 4:**
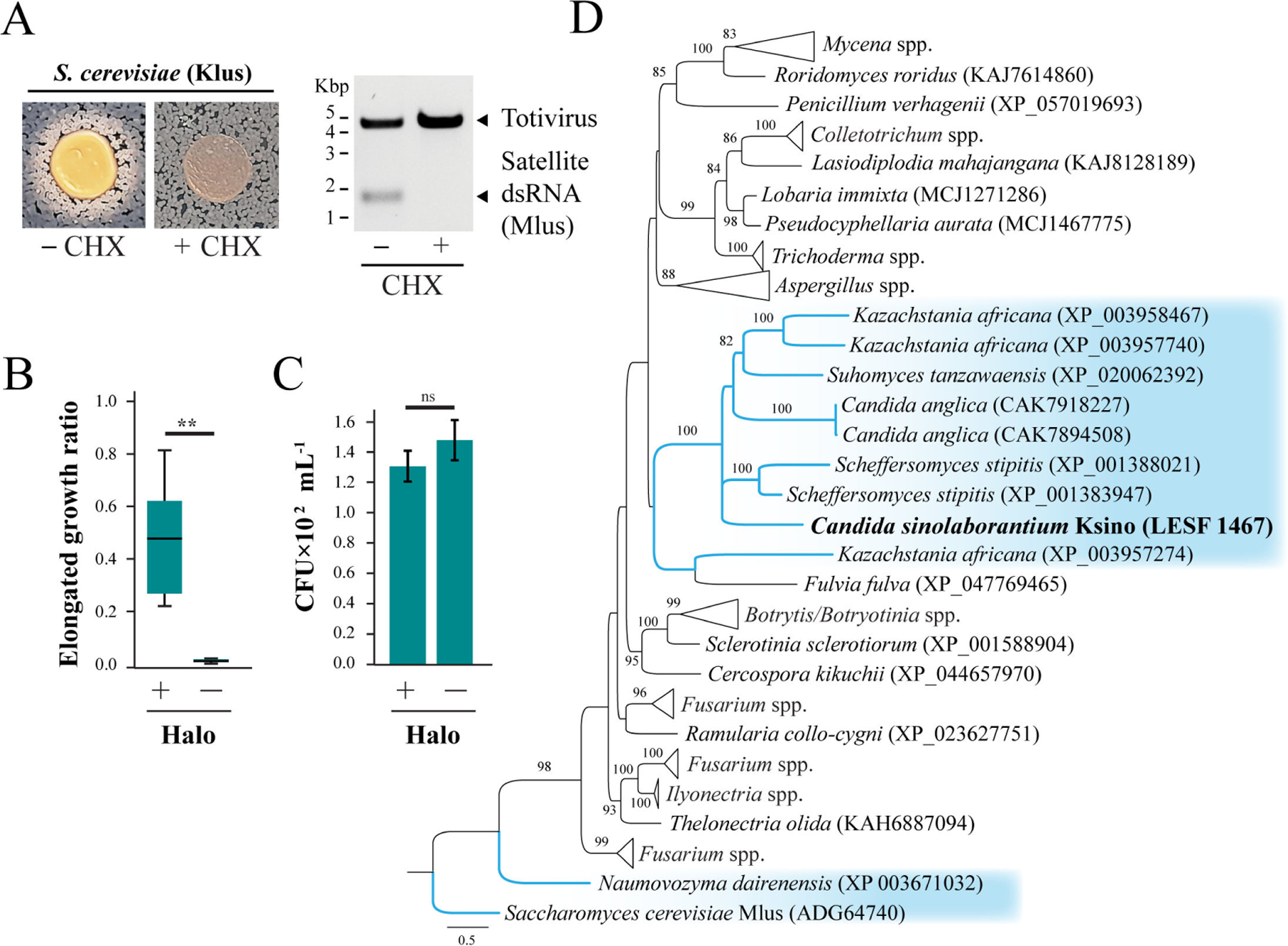
Ksino from *C. sinolaborantium* defines a novel group of killer toxins. (A) The phenotypes associated with the co-culture of the Klus-encoding killer yeast *S. cerevisiae* strain DSM-70459 with *K. ohmeri* (+Mlus). Treatment with cycloheximide (CHX) causes the loss of the Mlus satellite (−Mlus) and the loss of the white halo. The ratio of (B) elongated and (C) viable *K. ohmeri* cells in the white halo around Klus-producing *S. cerevisiae* (+Halo) and in axenic culture (−Halo). Asterisks indicate different means for Welch two-sample *t*-test (*P*-value < 0.01), ns = not significant. (D) An ML phylogeny of Ksino homologs identified in diverse fungi. Ksino from *C. sinolaborantium* is highlighted in bold, and all Saccharomycotina yeasts are highlighted in blue. The dsRNA-encoded killer toxin Klus is represented as an outgroup. Bootstrap values higher than 70 are shown. All error bars are standard error (*n* > 3).

The sequence similarity between Ksino and Klus prompted a more detailed investigation into whether these killer toxins share similar antifungal properties. Although we had previously failed to detect growth inhibition of *K. ohmeri* by *S. cerevisiae* killer toxin Klus (Fig. 2), we noted the appearance of white halos instead of a zone of growth inhibition or methylene blue staining after prolonged co-culture (Fig. 4A). The appearance of white halos was similar to those produced by *C. sinolaborantium* and was dependent on the presence of the Mlus satellite dsRNA that encodes the Klus killer toxin gene (Fig. 4A). Analysis of the cells within the white halo found a significant proportion of elongated cells, as previously observed upon exposure of *K. ohmeri* to *C. sinolaborantium* (Fig. 4B). However, unlike the Ksino halos, Klus did not cause a significant loss of cell viability (Fig. 4C).

To identify other possible Ksino-homologous proteins in different organisms, we performed a BLASTp search across the NCBI database, resulting in 84 protein sequences from various Ascomycota and Basidiomycota fungi. Ksino homologs were found in *Saccharomycetes, Pichiomycetes* yeasts, and other filamentous fungi with identity and alignment lengths higher than 30% and 109 amino acids, respectively (Fig. 4D; Table S11). Phylogenetic analysis of homologs, with Klus as an outgroup, indicates that proteins with the highest identity to Ksino (35%–42% identity) were all uncharacterized genes of the Saccharomycotina yeasts from the lineages of *Debaryomycetaceae* and the *Saccharomycetaceae* (Fig. 4D; Fig. S6 and Table S11). Saccharomycotina species with Ksino-like genes included *Candida anglica, Kazachstania africana, Scheffersomyces stipitis*, and *Suhomyces tanzawaensis*, with both *K. africana* and *S. stipitis* (Basionym: *Pichia stipitis*) that were previously reported as killer yeasts (83, 95). Filamentous fungi encode the majority of Ksino homologs (75 out of 84), including species belonging to the genera *Fusarium, Mycena*, and *Aspergillus*, known as plant and human pathogens (*Fusarium* spp.), saprotrophic and biotrophic plant-associated fungi (*Mycena* spp.), and fungi species that are ubiquitous in the environment (*Aspergillus* spp.) (96–101) (Fig. 4D).

**FIG 5:**
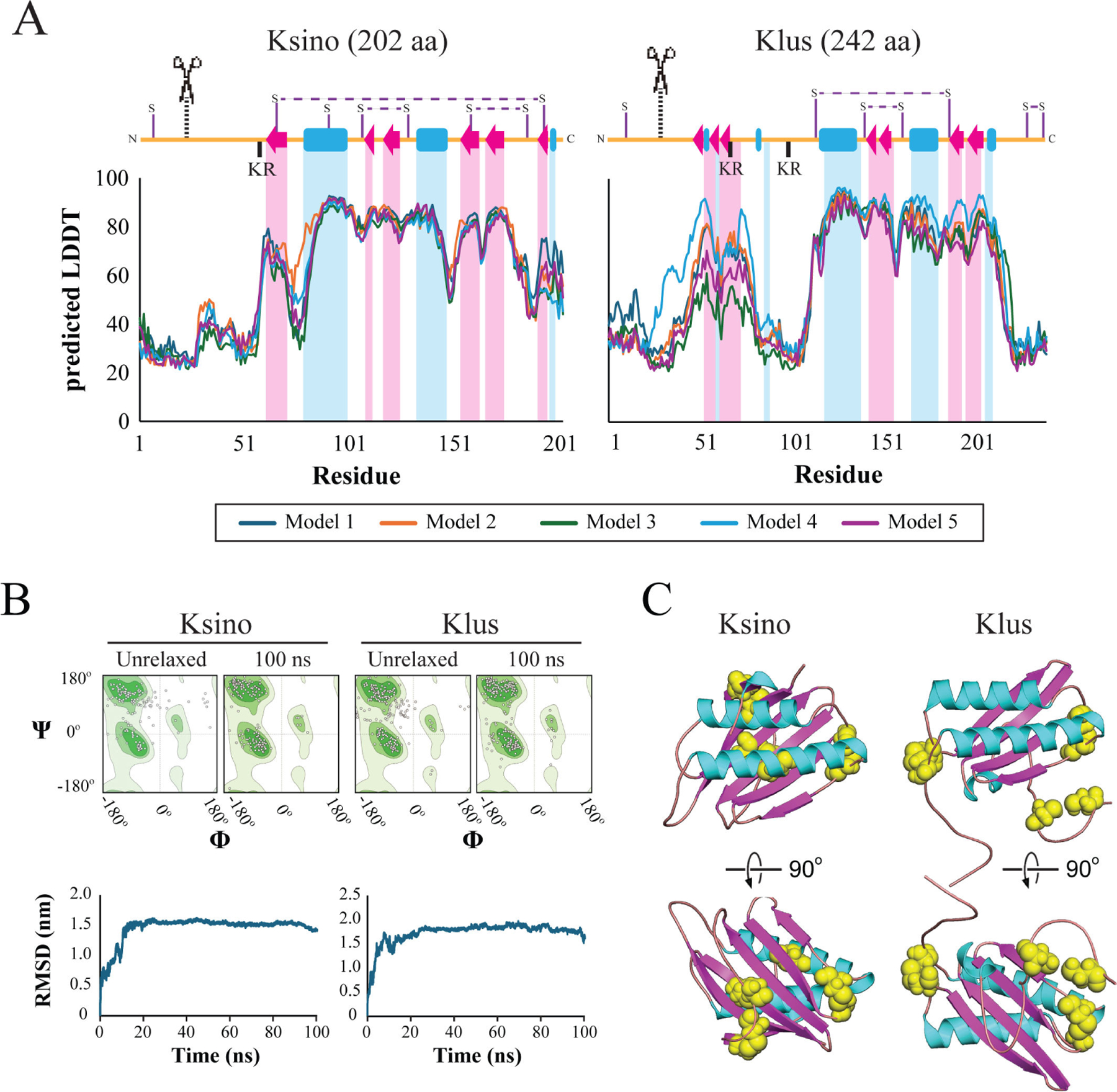
Molecular modeling of Ksino indicates structural homology to Klus. Predicted local distance difference test (pLDDT) confidence per residue and predicted disulfide bond arrangement of AlphaFold2 models of Ksino and Klus. (A) Secondary structure representations aligned with per-residue pLDDT scores from AlphaFold2. Alpha helices are represented as blue rectangles, beta sheets as red arrows, and unstructured regions as orange lines. Predicted disulfide linkages are indicated as horizontal dashed lines. Scissors with vertical dashed lines represent predicted signal sequence cleavage sites. (B) Ramachandran plots of general residues (non-proline/glycine) generated by SWISS structure assessment tool demonstrating improved Ramachandran favored after performing 100 ns simulation. The unrelaxed structure represents AlphaFold2’s raw output. (B) Full-protein RMSD over 100 ns molecular dynamics simulation. GROMACS was used to generate alignments of each snapshot back to the structure at 0 ns. (C) Ksino and Klus AlphaFold2 models with cysteine residues highlighted as yellow spheres to illustrate the predicted disulfide linkage arrangement. The cartoon helices and beta sheets are colored to match panel A.

The Ksino open reading frame was determined to be 202 amino acids long, comparable to Klus at 242 amino acids. Linear representation of the secondary structure revealed a similar pattern of alpha helices and beta sheets (Fig. 5A). There was also evidence of potential post-translational modification sites, including N-terminal signal sequence cleavage sites predicted by psipred (102) and a dibasic motif (i.e., KR) in the unstructured region before helix 1 (Fig. 5A). Dibasic motifs in killer toxins are known to be proteolytically cleaved by the Golgi-specific protease Kex2 (103). As Ksino has no sequence homology to a protein of known structure, AlphaFold2 was used to generate tertiary structure models of Ksino and Klus (104). Ksino and Klus had global local distance difference test (LDDT) scores of 63.6 and 65.1, respectively, and per residue, LDDT was greater than 80 in regions of the secondary structure for both proteins (Fig. 5A). Molecular dynamics simulation improved the bond angles of the AlphaFold2 model to Ramachandran favored regions in models of Ksino (77% to 91%) and Klus (73% to 89%) (Fig. 5B; Table S12). Ksino and Klus simulations reached a stable root mean square deviation (RMSD) of around 1.6 nm after approximately 20 ns of simulation (Fig. 5B). Molprobity scores, analogous to structure resolution, were determined to be 1.54 for Ksino and 1.86 for Klus (Table S12) (105). Overlay of the final energy-minimized structures of Ksino and Klus, compared with the original AlphaFold2 models, yielded an RMSD of 2.6 Å. The predicted organization of the Ksino structure consists of a discontinuous five-stranded antiparallel beta-sheet packed against a pair of antiparallel alpha helices (Fig. 5C). The tertiary structure of Klus follows the same overall organization but with one less beta-turn. Ksino has eight cysteine residues, compared to the seven in Klus. The predicted structures have three intramolecular disulfide bonds. In Ksino, C105–C127 is positioned to crosslink the C-terminus of helix 1 to the beta-sheet 2, similar to C141–C162 in Klus (Fig. 5C). The remaining predicted disulfide bonds are unique to both structural models. These simulations collectively suggest that the Klus and Ksino proteins share significant structural homology despite having low amino acid identity.

The molecular weight of Ksino was predicted to be 21.5 kDa, which appeared to be smaller than the predicted molecular weight determined by ultrafiltration (Fig. S5). To determine if Ksino was expressed by *C. sinolaborantium*, total RNA was extracted from laboratory-cultured yeast cells to assay for the presence of Ksino transcripts by RT-PCR.

**FIG 6:**
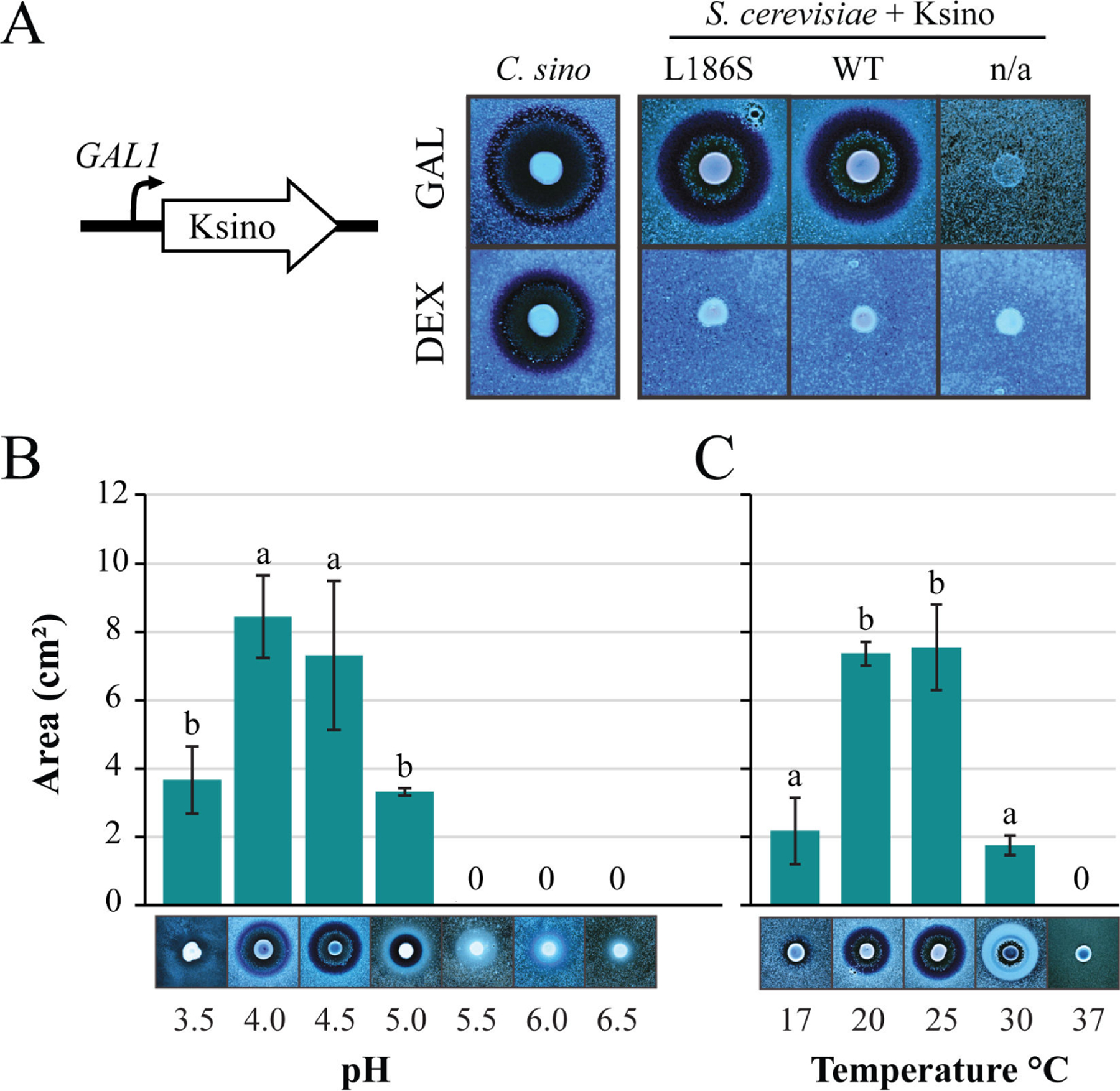
Ksino is an antifungal killer toxin when expressed by *S. cerevisiae*. (A) Recombinant expression of Ksino(L186S) by *S. cerevisiae* using galactose induction with a lawn of *C. castellii* Y-17070 (pH 4.5). Dextrose was used as a non-induced control. n/a indicates an empty vector negative expression control. Zones of growth inhibition were compared to the killer phenotype of *C. sinolaborantium*. Killer yeast activity under different (B) pH and (C) temperature conditions, as measured by the zone of growth inhibition and methylene blue staining. Representative images of the killer phenotype are displayed under each histogram. The means were significantly heterogeneous (one-way ANOVA, *P* < 0.01) for both the pH and temperatures tested. Means with the same letter are not significantly different from each other (Tukey–Kramer test, *P* > 0.05). All error bars are standard error (*n* = 3).

This approach successfully detected Ksino expression under laboratory conditions by the presence of Ksino mRNA (Fig. S7). To confirm that Ksino is an active killer toxin, the gene was amplified by PCR and cloned into a *S. cerevisiae* expression vector under the control of a galactose-inducible promoter. *C. sinolaborantium* belongs to the Serinales order and uses the CUG codon to encode serine (106, 107). Ksino has one CUG codon that introduces a leucine at position 186 when expressed in *S. cerevisiae*. This mutation is predicted to have a small destabilizing effect on folding compared to the wild-type based on FoldX 5.0 and molecular dynamics simulation (estimated ΔΔG_Folding_ = 1.0 kcal/mol) (Fig. S8). Therefore, residue 186 was recoded for serine in *S. cerevisiae* (L186S). When grown on galactose-containing media, no growth inhibition of *K. ohmeri* was observed during co-culture with a laboratory strain of *S. cerevisiae* transformed with the wild-type Ksino or Ksino(L186S) expression vectors (Fig. S9). However, challenging the strain *C. castellii* Y-17070 with *C. sinolaborantium* killer yeast or Ksino(L186S) expressed by *S. cerevisiae* on galactose triggered large zones of inhibition and methylene blue staining, indicating that Ksino is a novel killer toxin (Fig. 6A). Growth of *S. cerevisiae* under different temperatures and pH found that Ksino has an optimum antifungal activity between pH 4 and 4.5 (Fig. 6B; Table S13) and 20°C–25°C (Fig. 6C; Table S14), which was narrower than the killer phenotype of *C. sinolaborantium* (Fig. 3). The inability of Ksino expressed by *S. cerevisiae* to prevent the growth of *K. ohmeri* and differences in the optimal conditions for antifungal activity against *C. castellii* indicated that Ksino may be one of several antifungal molecules produced by *C. sinolaborantium* or that the expression of Ksino by *S. cerevisiae* alters its spectrum of activity.

## DISCUSSION

The large number of killer yeasts in fungicultures suggests they might play a role in attine ant fungus gardens by suppressing fungal competitors by allelopathic effects on the complex dynamics of microbial interactions. Different studies have revealed that yeasts are abundant in attine ant environments that likely represent differences in the foraging composition and substrate preparation behaviors observed among the different fungicultures (45, 108, 109). Yeasts from fungus gardens of the leaf-cutting ant *Atta texana* were observed to suppress the growth of fungal garden contaminants, especially *Escovopsis* and *Syncephalastrum* (44). These antifungal properties were hypothesized to contribute to fungal garden “immunity,” whereby the resident fungi within a fungiculture prevent invasion by other fungal species. This would require interplay between the cleaning behaviors of attine ants in their gardens to remove unwanted fungi and other microbial interactions within attine ant colonies, e.g., allelopathy. Our study indicates that fungus gardens of attine ants harbor killer yeasts with similar prevalence to other surveys (10–17). This expands the previous findings regarding the prevalence of killer yeasts in laboratory-reared attine ant colonies of *Atta sexdens* (47). Moreover, the prevalence of killer yeasts among the different ant species, such as *Mycetophylax* aff. *auritus* (43%, eight nests sampled) and *Mycetomoellerius tucumanus* (46%, four nests sampled), was considerably higher than in other surveyed fungicultures.

Killer yeasts from attine ant colonies were more likely to inhibit the growth of susceptible strains from different fungicultures. This supports previous observations of killer and non-killer yeast interactions, whereby local selective pressure for killer toxin resistance allows the coexistence of killer and non-killer yeasts in a given niche (12, 13, 15, 54). The susceptibility of yeast populations that have never been exposed to a specific killer toxin would depend on the standing genetic variation (perhaps in genes involved in cell wall biogenesis and membrane toxin receptors) among different lineages of yeasts, dictated by the population’s evolutionary history. Our findings support the hypothesis that yeasts contribute to fungal garden “immunity” since killer yeasts are expected to prevent the invasion of other fungal species into the fungus garden. The coexistence of killer and non-killer yeast species could be an outcome of microhabitats in the spatially structured environment of the fungus garden, which may harbor different populations of yeasts and allow colonization by killer yeasts (50, 60).

Saccharomycotina yeast species isolated in attine ant gardens have been previously found to secrete killer toxins. Among the yeast species, the ability to produce toxins was observed for *Diutina catenulata* (Basionym: *Candida catenulata* [110, 111]), *Kodamaea ohmeri* (Synonym: *Pichia ohmeri* [112–114]), *Schwanniomyces vanrijiae* (115), and *Wickerhamomyces ciferrii* (Basionym: *Hansenula ciferrii* [116]). The exception was *Jamesozyma spencerorum* (Synonym: *Kazachstania spencerorum* nom. inv.), which, to our knowledge, is the first report of a killer phenotype displayed by this species, although toxin production has been previously reported in the genus, specifically *K. africana*, *K. exigua*, and *K. unispora* (83, 110, 117). These yeast species have also been identified in substrates that are commonly foraged or associated with attine ants, including plants (*Schwanniomyces vanrijiae* [115]; *Kodamaea ohmeri* [112]), insects and their environments (*Candida blattae* [118]; *Candida sinolaborantium* [119, 120]; *Schwanniomyces vanrijiae* [121]; *Kodamaea ohmeri* [119, 122, 123]), and soil (*Jamesozyma spencerorum*, Synonym: *Kazachstania spencerorum* nom. inv (124–127).

Importantly, killer yeast species appear to be unique between colonies of the same ant species (41). This observed diversity could be due to the foraging behavior of ants, whereby sampling of the surrounding environment would introduce different fungal species to the fungiculture of the same species of ants in different geographical locations (41). However, despite the potential role of killer yeasts in garden immunity, whether the occurrence of killer yeasts in a fungiculture is acquired by chance, inherited from the environment, or inherited during garden propagation remains unknown (45). Niche construction by the fungal cultivar species could also play a role in yeast diversity in the fungus garden environment or by other features such as the pH of the fungus garden. Interestingly, the pH observed in the different fungus gardens (pH 5.1–5.4) is consistent with the optimal antifungal activities of *C. sinolaborantium* (pH 3.5–5.0) and Ksino (pH 4.0–4.5). However, the association between killer yeasts and fungicultures still requires robust empirical evidence to reject the stochastic nature of killer yeast colonization. Future yeast isolation will provide new directions to clarify whether killer yeast occurrence is modulated by ant foraging choice or fungus garden features such as pH and fungal cultivar species.

Our survey of the encoding elements of killer toxins indicated that the 180 isolated strains of Saccharomycotina yeasts were devoid of cytoplasmic killer toxin-encoding elements such as dsRNA satellites and dsDNA linear plasmids. The frequency of dsRNA elements associated with *Saccharomyces* yeasts has been observed to range from 10% to 51% in wild and domesticated strains (77, 128). It is expected that more of these genetic elements will be found in other Saccharomycotina lineages, given their discovery in non-*Saccharomyces* yeasts and the frequency of genome-integrated molecular fossils of plasmid and virus sequences in different lineages (86, 90, 129–133). Most of the yeasts isolated from fungicultures belong to the Serinales, where the CUG codon is translated into serine instead of leucine in the universal genetic code (106). The rewiring of the fungal genetic code has been suggested to prevent the invasion of viruses and plasmids by stopping the faithful translation of viral proteins (107). Alternatively, RNA interference (RNAi) might also contribute to limiting viral infections in these yeasts, even though, in some cases, yeast lineages maintain viruses that suppress RNAi (134–138). RNAi and genome recoding could be possible reasons for the absence of cytoplasmic elements in the yeasts associated with attine ant fungiculture. However, this still requires a more in-depth investigation to exclude the stochastic nature of virus acquisition by fungi and sampling biases.

The lack of extrachromosomal elements in killer yeasts associated with attine ant fungiculture suggests that killer toxin genes are genome-encoded. Our survey indicates that killer toxins from different Saccharomycotina lineages associated with fungus gardens differ in their antifungal spectra from canonical *Saccharomyces* killer toxins (K1, K2, K28, etc.). In particular, toxins secreted by *C. sinolaborantium* had the broadest activity, including toward infectious human pathogens (e.g., *Candida albicans, C. glabrata*, and *C. auris*). A unique response to the killer activity of *C. sinolaborantium* was also observed in *K. ohmeri*, where cells exposed to the killer yeast grew as filaments. Usually, dimorphism in yeasts has been demonstrated to be triggered by nitrogen starvation (139), different carbon sources, and temperature (140). However, to our knowledge, this is the first case of this phenotype associated with exposure to a killer yeast. The broad spectrum antifungal activities of *C. sinolaborantium* and the ability to fractionate antifungal activities by ultrafiltration indicate the likely presence of multiple unidentified chromosomally encoded toxins. After determining the genomic sequence of *C. sinolaborantium*, the new killer toxin Ksino was discovered due to its sequence homology to the *Saccharomyces* killer toxin Klus. However, the killer activity of *C. sinolaborantium* was not fully reproduced when Ksino was expressed by *S. cerevisiae* and was unable to inhibit or cause the elongated growth of *K. ohmeri*. Instead, robust growth inhibition was only observed after challenging *C. castellii*. These data also support the expressions of multiple killer toxins by *C. sinolaborantium* or that ectopic heterologous expression by *S. cerevisiae* is inadequate due to potential deficiencies in extracellular export or protein posttranslational modification.

Many proteins from different Ascomycete and Basidiomycete fungi lineages were identified in our search for Ksino-like proteins, including filamentous fungi and yeast (from Saccharomycetes and Pichiomycetes). The Ksino-like proteins from yeasts of the Saccharomycotina subphylum (*Candida anglica, Kazachstania africana, Scheffersomyces stipitis*, and *Suhomyces tanzawaensis*) share a closer evolutionary origin to Ksino and therefore might represent bona fide killer toxins. Despite our approach to identifying killer toxins being limited by primary sequence similarity to known toxins, it has successfully identified Ksino. This combination of genome sequencing, sequence homology searches, and structural modeling could be used more widely on the many killer yeasts that are devoid of cytoplasmic extrachromosomal elements to identify new killer toxins.

In summary, this study demonstrates that (i) fungus gardens of attine ants can harbor killer yeasts, an underappreciated environment for killer yeasts. (ii) DsRNA and dsDNA extrachromosomal elements are lacking from yeasts associated with attine ant fungicultures. (iii) Genome mining can discover new chromosomally encoded killer toxins, specifically Ksino secreted by *C sinolaborantium*. (iv) Ksino has sequence and structural homology to the *Saccharomyces* killer toxin Klus and is a member of a new family of killer toxins in Ascomycete and Basidiomycete fungi.

## MATERIALS AND METHODS

### Yeast source and phylogenetic analysis

To investigate the prevalence of killer yeast in the fungus garden of attine ants, a representative collection of 180 yeasts was examined (41) (Table S1). Briefly, yeasts were obtained by fungus garden suspension in saline solution (0.85% NaCl) supplemented with 0.05% Tween 80. Suspensions were tenfold-diluted and surface-spread on nutrient media (yeast-malt agar and Sabouraud dextrose agar, supplemented with 150 μg mL^−1^ chloramphenicol, and pH adjusted to 4.5; detailed in 41). Yeast characterization involved microsatellite amplification analysis (MSP-PCR [141]) and sequencing of the D1/D2 region of the large subunit ribosomal RNA gene (142). Our selection of the 180 *Saccharomycotina* yeasts spanned from fungus gardens of the leaf-cutting ant *Acromyrmex coronatus* (*n* = 91), the non-leaf-cutting ant *Mycetomoellerius tucumanus* (*n* = 13), the lower attines *Mycetophylax* aff. *auritus* (*n* = 42) and *Mycocepurus goeldii* (*n* = 25), which cultivate *Agaricaceae fungi*, and *Apterostigma goniodes* (*n* = 9), which cultivates *Pterulaceae* fungi.

To assess the phylogenetic relationship between the yeast strains, we performed a phylogenetic analysis with representative species and their closest relatives (the accession number of sequences used in phylogenetic analysis is listed in Table S2). Sequences were aligned with MAFFT v.7 (143), and nucleotide substitution models were generated using the Bayesian information criterion (BIC) by the standard model generation in IQ-TREE2. SYM+FQ+I+G4 was selected as the nucleotide substitution model. ML phylogenetic trees were reconstructed with ultrafast bootstrap in IQTREE2 (144), with 10,000 replicates for ultrafast bootstrap (tree completed after 262 iterations, LogL: −14,973.244). The final trees were edited in FigTree v.1.4.3.

### Killer yeast assays

Killer assays were performed to confirm killer toxin production by yeasts, as described in 88. The 180 yeast strains were seeded onto yeast-peptone-dextrose (YPD) 4.6 dextrose “killer assay” agar plates (yeast extract 10 g L^−1^, peptone 20 g L^−1^, dextrose 20 g L^−1^, sodium citrate 29.9 g L^−1^, supplemented with 0.003% wt/vol methylene blue, and pH adjusted to 4.6 with HCl), seeded 10^5^ cells mL^−1^ with one of the 49 selected yeast-susceptible strains (i.e., from fungus gardens and sources not related to the attine ant environment, Table S15). Nine *Saccharomyces* spp. strains that encode known toxins were used as positive controls for killer toxin production, including K1, K1L, K21, K2, K28, K45, K62, K74, and Klus (Table S15). Yeasts were also evaluated using 20 pathogenic yeasts as susceptible strains (Table S15). Toxin production was recorded after 5 days of incubation at RT, either by an inhibition zone or a methylene blue stain of susceptible strains. Killer activity severity was scored following Fredericks et al. (83) by zone of inhibition with or without methylene blue halo or by strong or weak methylene blue halo without inhibition. We applied heatmaps to visualize the species interactions, employing the function heatmap.2 from the gplots package, with the default configuration for dendrogram display (145). To evaluate the effect of the yeast sources (i.e., same or different fungicultures or sites) on killer toxin activity (i.e., killed or nonkilled). We selected 9,000 interactions (toward 50 strains that showed susceptibility to killer toxins) and analyzed them with Pearson’s chi-squared test. All yeasts used in killer assays are listed in Table S15.

### Extraction of dsRNA-encoding elements

To determine if the yeast strains host dsRNA elements, we extracted the dsRNA elements from all yeast strains following Okada et al. (146), with modifications as stipulated in Crabtree et al. (88). The K1 killer yeast *S. cerevisiae* BJH001 was used as a positive control for all assays. Specifically, yeast cultures were grown in YPD: yeast extract 10 g L^−1^, peptone 20 g L^−1^, and dextrose 20 g L^−1^), centrifuged at 3,000 × *g* for 5 min, and the broth was removed by aspiration. Cells were resuspended in water, centrifuged at 8,000 × *g*, and aspirated. A total of 450 μL of 2× sodium chloride-Tris-EDTA buffer (STE: 200 mM NaCl; 20 mM Tris-HCl, pH 8.0; 30 mM EDTA) and 0.5 μL β-mercaptoethanol were added, and cells were disrupted in a cell disruptor (Digital Disruptor Genie, Scientific Industries) for 3 min at 3,000 rpm. Then, 50 μL of 10% (wt/vol) sodium dodecyl sulfate (SDS) solution and 350 μL phenol–chloroform–isoamyl alcohol (25:24:1) were added to crude extracts, which were homogenized and centrifuged at 10,000 × *g* for 5 min. Then, 500 μL of the supernatant was mixed with absolute ethanol (5:1), and the final volume of 600 μL was transferred to cellulose columns (i.e., 0.05 *g* cellulose D in a 0.6 mL tube bottom punctured and placed in collecting tubes).

Column tubes were centrifuged at 10,000 × *g* for 30 seconds, and the flow-through was discarded. Then, 350 μL of wash buffer (1× STE containing 16% vol/vol EtOH) was added to the column, and the tubes were centrifuged at 10,000 × *g* for 30 seconds, followed by discarding the flow-through. Columns were transferred to a collecting tube, 350 μL of 1× STE was added, and the tubes were centrifuged at 10,000 × *g* for 30 s. The eluted fraction was recovered, and 1 mL of absolute ethanol with 40 μL of 3 M aqueous sodium acetate (pH 5.2) was added. Tubes were inverted ten times and centrifuged at 20,000 × *g* for 15 min. The ethanol mix was aspirated without disturbing the precipitated pellet, which was then air-dried and dissolved in 15 μL of molecular-grade water. The presence of dsRNAs was examined (loading 5 μL of each sample) by 0.8% TAE agarose gel electrophoresis, stained with ethidium bromide, at 130 V for 30 min in 1% TAE buffer. As a molecular weight reference, a 1 kb DNA ladder was used. The *Saccharomyces cerevisiae* strain YJM1307, which harbors dsRNA totiviruses and satellites, was used as a positive control.

### Extraction of the dsDNA linear plasmid

To determine if dsDNA linear plasmids encode the killer toxins, we extracted the dsDNA elements from positive killer yeast, *Kluyveromyces lactis* AWJ137 with the linear DNA plasmids pGKL2 (13.5 kb) and pGKL1 (8.9 kb) and *Pichia acaciae* NRRL Y-18665 with the linear plasmids pPac1-1 (12.6 kb) and pPac1-2 (6.8 kb) (147, 148). Cultures were grown in YPD overnight, 200 μL was centrifuged at 3000 × *g* for 5 min, and the broth was removed by aspiration. Yeast cells were resuspended in 10 μL zymolase solution (5% of 50 mM Tris-HCl, pH 8; 10% of 5 mM EDTA, and 85% water) and 7.2 μL of 10 mg mL^−1^ of zymolase (United States Biological Life Sciences, Swampscott, MA), and the suspension was incubated at 30°C for 1 h. After incubation, 1 μL of 10% SDS and 3 μL of proteinase K (New England Biolabs, Ipswich, MA) were added to the suspension, followed by an incubation of 1 h at 65°C. After incubation, 5 μL of the loading dye was added, and the suspension was centrifuged at 3,000 × *g* for 5 min. The presence of dsDNA plasmids was examined (loading 10 μL of each sample) by 0.6% TAE agarose gel electrophoresis, stained with ethidium bromide, at 130 v for 1 h in 1% TAE buffer.

### Toxin precipitation and killer toxin activity at different temperatures and pH

To investigate the stability of the toxins produced by *C. sinolaborantium* LESF 1467, the isolate extracts on YPD medium (pH 4.6) were filtered, mixed with 1:1 absolute ethanol, centrifuged at 20,000 × *g* for 20 min, followed by supernatant aspiration and suspension in 10 μL of YPD (pH 4.6). To confirm the killer activity of the precipitates, 4 μL of the suspended proteins was used in killer assays against *Kodamaea ohmeri* LESF 1474. To evaluate the thermostability, the precipitated toxins were screened in killer assays incubating at RT or heat-inactivated at 60°C and 98°C for 2 min. For quantitative analysis of killer toxin expression, 2 μL of 10^8^ cells mL^−1^ was plated onto susceptible lawns. The killer activity was also evaluated for activity at different pHs, adjusting the pH to 3.5, 4, 4.5, 5, 5.5, and 6 with HCl 12N, and at various temperatures, incubating at 17°C, 20°C, 25°C, 30°C, and 35°C. The area of activity on killer assays (i.e., zone of inhibition and cell differentiation of the lawn strain) was measured in cm^2^ after 4 days with ImageJ 1.53t (149). Since the area data do not satisfy the assumptions of parametric tests, we analyzed variance with Kruskal-Wallis followed by the Wilcoxon rank sum test with Bonferroni correction as a post hoc for pairwise multiple comparisons. Analyses were performed using the software RStudio v. 2023.12.0.369 (POSIT TEAM, 2023) and R v. 4.3.2 (R CORE TEAM, 2023). Figures were prepared using ggplot2 (150).

### Production of elongated cells by *Kodamaea ohmeri* against killer toxins

We observed that the yeast *K. ohmeri* LESF 1474 produces elongated cells in the presence of *C. sinolaborantium* toxins and their precipitates. We suspended the cells in the white and raised zones of killer assays and on axenic cultures in 1× PBS. Cell morphology was checked under a microscope (Zeiss Primo Star iLED) by counting budding (2 to 17 × 10^6^ cells mL^−1^) and elongated cells (2 to 15 × 10^5^ cells mL^−1^) with a Neubauer chamber. Cell survival was evaluated by adjusting the suspensions to 10^3^ cells mL^−1^ and seeding 100 μL in YPD. CFUs were counted after 3 days of incubation at RT. A similar elongated cell phenotype was observed in the presence of *S. cerevisiae* DSM-70459, which secretes the known killer toxin Klus (encoded by dsRNA elements). Microscopic and survival evaluations were also conducted in the presence of *S. cerevisiae* DSM-70459, as described above. To confirm that the elongated cell phenotype is related to the presence of the dsRNA satellites, dsRNA satellites were cured from *S. cerevisiae* by growing on a YPD medium supplemented with 5.0 μM cycloheximide. Loss of killer toxin production and the loss of dsRNAs were assayed as described above. The ratio between normal and elongated cells was compared between *K. ohmeri* in the white halo around *C. sinolaborantium* and *S. cerevisiae* DSM-70459 with axenic cultures surrounding the halo, with ten independent replicates. The data were checked for the assumption of parametric tests (performing Shapiro-Wilk and Bartlett tests, respectively, for normality and homogeneity of variances). The ratio data in both treatments, toward *C. sinolaborantium* and *S. cerevisiae* DSM-70459, were found not to satisfy the assumptions of equal variance (Bartlett test, df = 1, *P*-value < 0.001), so we conducted the Welch Two Sample *t*-test to compare means between treatments. The same procedure was performed for cell survival data toward *C. sinolaborantium*, which also did not satisfy the assumptions of equal variance (Bartlett’s K-squared = 4.16, df = 1, *P*-value = 0.04). Cell survival data toward *S. cerevisiae* DSM-70459 satisfied the assumptions for parametric tests (Bartlett’s K-squared = 0.52, df = 1, *P*-value = 0.47; Shapiro-Wilk *P*-value > 0.05), so we conducted the Two Sample *t*-test to compare means between treatments.

### Genomic DNA extraction, sequencing, and annotation

To extract genomic DNA from *C. sinolaborantium*, the yeast was grown in YPD broth; cells were recovered by centrifugation (3,000 × *g* for 5 min), washed, and suspended in 500 μL TE buffer (10 mM Tris-Cl; 0.1 mM EDTA, pH 8). Recovered cells were transferred to a microtube, centrifuged as previously described, and the supernatant was removed. The pellet was disrupted by vortexing. Cells were suspended in a lysis buffer (2% Triton X-100, 1% SDS, 100 mM NaCl, 10 mM Tris-HCl, pH 8, 1 mM EDTA pH 8) with approximately 300 μL of glass beads. A volume of 200 μL of phenol-chloroform-isoamyl alcohol solution was added, and the sample was vortexed for 3 min at 3,000 rpm (Disruptor Genie), followed by the addition of 200 μL of TE buffer and centrifugation for 5 min at 21,000 × *g*. The upper layer was transferred to a new microtube and mixed by inversion with 1 mL of absolute ethanol. The tube was centrifuged for 3 min at 21,000 × *g*, the supernatant removed, and the pellet suspended in 400 μL of TE buffer. Then, 30 μL of DNase-free RNAse A was added, and the solution was mixed and incubated for 5 min at 37°C. The DNA was precipitated by adding 10 μL of 4 M ammonium acetate and 1 mL of absolute ethanol, mixing by inversion, and centrifuging for 3 min at 21,000 × *g*. The supernatant was discarded, and the pellet was dried for approximately 20 min at RT. Genomic DNA was suspended in 100 μL of TE buffer and stored at −20°C.

The genome was sequenced by Illumina NextSeq 550 using the paired-end method. Libraries were created with the Illumina DNA Prep kit for whole-genome sequencing (WGS) (https://www.illumina.com/products/by-type/sequencing-kits/library-prep-kits/nextera-dna-flex.html). The initial quality analysis and cleaning steps were performed with fastp v. 0.20.1(151). The genome was assembled with SPAdes v. 3.15.5 (152) with multiple k-mers (21, 31, 41, 51, 61, 71, and 81). The blobtools v. 3.5.2 software was used to check contaminants. Briefly, Blastn 2.13.0+ was used to search for structures across the entire NCBI nt database; minimap2 v. 2.24-r1122 (22) and samtools v.1.13 were used to obtain sequencing coverage (153–155). The blobtools visualization was used to ascertain the results. The rDNA and internal transcribed spacer (ITS) regions were annotated with ITSx v. 1.3, which was compared to an amplicon previously submitted to NCBI for this isolate (accession: ON493969) as part of the contamination verification steps (156). Final assembly statistics were obtained with QUAST v. 5.2.0 (157). Completeness was checked using BUSCO v. 5.4.4 (93) with the saccharomycetes_odb10 data set (2,137 BUSCOs). The genetic prediction was performed using Braker v. 2.1.6 [which includes Augustus v. 3.4.0, ProtHint v. 2.6.0 and GeneMark-ES v. 4.71] with OrthoDB v. 10 methods (158–161). InterProScan v. 5.60 was used for functional annotation of predicted proteins (162).

### Genome mining for toxin-encoding regions

To identify toxin-encoding chromosomal regions, a search using proteins predicted from WGS was performed with BLASTP 2.9.0, using a protein sequence database of known killer toxins. Results with query/hit alignments greater than 100 were selected. An open reading frame similar to the Klus toxin (28% identity, with 151 aa alignment length and an e-value of 1.56E^−05^) was found and selected for further analysis. PCRs were performed to amplify the region of interest using primers designed with Primer3 software 4.1.0 (https://primer3.ut.ee/). The primers were designed to target Ksino untranslated regions (961F: 5′-TTAACGACTTTCGTCTTCGCTATCC-3′ and 961R: 5′-ATTGAGATCAGGTGGCCTGTGTAGC-3′) followed by nested-PCR to amplify the open reading frame using the previous PCR product as the template (961NF: 5′-CGATCACCTAGCCCAAAATGC-3′ and 961NR: 5′-AAAGTGTTGGCCAAGGACACG-3′). Both of the amplification reactions used Phusion high-fidelity master mix (New England Biolabs, Ipswich, MA) with the amplification conditions of 98°C for 3 min, 30 cycles at 98°C for 30 s, 63°C for 30 s, 72°C for 30 s, and a final extension step at 72°C for 5 min. Amplicons were sequenced to confirm the identity of the region of interest.

### Prediction of the tertiary structure and comparative analysis of proteins

After confirming the genomic coding region of Ksino was the correct size using PCR, the sequence was used to predict the protein structure via the AlphaFold2 software with default parameters (104). The B factor columns of each unrelaxed structure were used to create per residue pLDDT graphs. The secondary structure was assigned using PyMol to create a linear representation of the protein and aligned to per residue graphs. The top-scoring relaxed model from AlphaFold2 was used for molecular dynamics simulation using the GROMACS package (163). Briefly, the system was set up using the AMBER99SB-ILDN force field with TIP3P water, a dodecahedral box with 1.0 nm padding, and 0.15 M NaCl. The system was then subjected to energy minimization by steepest descent, followed by NVT and NPT equilibration with a pressure of 1.0 atm and temperature of 300 K. Pressure and temperature were maintained constant using Parrinello-Rahman with isotropic coupling and V-rescale with protein non-protein as the coupling groups, respectively. This setup was chosen to match standard cellular conditions closely: 26.85°C, 0.15 M NaCl, pH 7, and 1 atm. Simulations were run for 100 ns with a 2.0 fs timestep, and snapshots were saved every 2 fs. The RMSD was calculated as a function of time using the resulting trajectories. In addition, the final snapshot of each trajectory was saved as a Protein Data Bank structure file and compared to relaxed and unrelaxed AlphaFold2 models using the SWISS structure assessment tool (105).

FoldX was used to predict the ΔΔG_Folding_ of L186S (164). Amber relaxed Ksino L186 was used as the starting structure, and FoldX PDBrepair was carried out six times to optimize the structure. Then, L186 in the optimized structure was mutated to every other residue using PositionScan. ΔΔG_Folding_ was taken from the output file.

### Bioinformatic discovery of Ksino homologs in fungi

A search for similar sequences in NCBI (https://www.ncbi.nlm.nih.gov/) was also carried out with Blastp, with the results filtered by alignment length (above 109), identity (above 30%), and e-value (less than 0.03). The protein sequences were aligned with MUSCLE (https://www.ebi.ac.uk/jdispatcher/msa/muscle), with the alignment consisting of 86 sequences and 426 positions. Amino acid substitution models were generated according to the Bayesian information criterion (BIC), with WAG+R4 being the chosen model. ML phylogenetic trees were reconstructed in IQTREE2, with 10,000 ultrafast bootstrap replicates (tree completed after 163 iterations, LogL: −18,525.485). The final trees were edited in FigTree v.1.4.3.

### Cloning and transformation in *Escherichia coli*

To study the coding region, a “TA” terminal was added to the amplicon in a reaction that included 1 μL Taq polymerase, 19 μL of the PCR product, 1 μL of 10 μM dNTPs, 5 μL of 10× buffer, and 24 μL of sterile ultrapure water. The reaction was incubated at 72°C for 20 min. For cloning, the pCR8/GW/TOPO TA Cloning kit (Invitrogen, Waltham, MA) was used, adding 1 μL of amplicons with TA terminals, 0.25 μL of saline solution from the kit, and 0.25 μL of the pCR8/GW/TOPO vector. The reaction was incubated at RT for 2 h, followed by adding 25 μL of competent *E. coli* cells (One-Shot TOP10 Competent Cells, Invitrogen, Waltham, MA). The reaction was kept on ice for 30 min, followed by heat shock at 42°C for 30 s and then in an ice bath for 2 min. Subsequently, 250 μL of Super Optimal broth (SOC: 20 g L^−1^ tryptone, 5 g L^−1^ yeast extract, 0.58 g L^−1^ NaCl, 0.19 g L^−1^ KCl, 2.03 g L^−1^ MgCl_2_, 1.20 g L^−1^ MgSO_4_, and 3.6 g L^−1^ dextrose) preheated to 37°C was added and incubated at 37°C for 1 h. Then, 250 μL was plated on Luria-Bertani medium (LB: 10 g L^−1^ of tryptone, 5 g L^−1^ of yeast extract, 10 g L^−1^ of NaCl, and 15 g L^−1^ of agar) supplemented with 100 μg mL^−1^ of spectinomycin. Plasmids were extracted using the High-Speed Mini Plasmid kit (IBI Scientific, Dubuque, IA), following the manufacturer’s recommendations.

Due to the transcriptional diversity of CUG-Leu codons in some yeasts, the vectors containing the region of interest were subjected to targeted mutations (Site-Directed Mutagenesis, SDM) in a CUG codon starting at position 556 of the DNA sequence (position 186 in the protein sequence). Thus, the mutants consist of the alternatives identified for CUG in other yeasts, being called L186A and L186S, with L being the original amino acid, 186 the position, and “S” the mutation for Ser. To obtain the mutants, we design primers targeting position 186 as follows: 961GCG: 5′-CAGCTGTGGCGCGAACCATAGCGGG-3′, 961TCG: 5′-CAGCTGTGGCTCGAACCATAGCGGG-3′ (forward), and 961SNM: 5′-AGTCCCGCAGGCCAATCC-3′ (reverse). Amplification reactions included 12.5 μL of Phusion High-Fidelity Master Mix, 1 μL 10 μM forward primer, 1 μL 10 μM reverse primer, 9.5 μL sterile ultrapure water, 1 μL 1:9 diluted DNA (i.e., pCR8 plasmid with the region of interest). Amplification conditions were as follows: 98°C for 30 min, 25 cycles at 98°C for 5 s, 67°C for 10 s, 72°C for 50 s, and a final extension step at 72°C for 5 min. To remove the template DNA from the reaction, a Dpnl treatment was performed by mixing 22 μL of the PCR product, 2 μL Dpnl, 3 μL CutSmart buffer, and 3 μL sterile ultrapure water. The reaction was incubated at 37°C for 1 day. For the addition of 5′-P and 3′-OH, we performed a PNK treatment: 30 μL of the treated product, 5 μL PNK buffer, 1 μL PNK, 5 μL 10 mM ATP, 1 μL 0.1 M DTT, and 8 μL of sterile ultrapure water. The reactions were incubated for 30 min at 37°C and inactivated at 65°C for 10 min. The product was purified using the Monarch DNA & PCR Cleanup kit (New England Biolabs, Ipswich, MA), following the manufacturer’s protocols. To re-ligate the plasmids, the following reaction was performed: 2 μL of purified product, 1 μL T4 DNA ligase, 2 μL 10× binding buffer, and 15 μL sterile ultrapure water. The reaction was incubated for 2 h at RT, and then 25 μL of competent *E. coli* cells (One-Shot TOP10 Competent Cells, Invitrogen, Waltham, MA) was added. Transformed cultures were selected in LB supplemented with 100 μg mL^−1^ spectinomycin, plasmids extracted with the High-Speed Mini Plasmid kit (IBI Scientific, Dubuque, IA), and mutations checked by sequencing (ELIM Biopharmaceuticals, Hayward, CA).

The vectors were subjected to a Gateway recombination from the vectors in pCR8 to pAG306-GAL-ccdB. For recombination, the reaction was conducted with 0.5 μL of the source vector, 0.5 μL of the destination vector, 1 μL of sterile ultrapure water, and 0.5 μL of LR Clonase II (New England Biolabs, Ipswich, MA). The reaction was incubated for 4 h at RT, followed by adding 0.25 μL of Proteinase K (New England Biolabs, Ipswich, MA) and incubating at 37°C for 10 min. Finally, the *E. coli* transformation was performed as previously described, and cultures were selected in LB supplemented with 100 μg mL^−1^ of chloramphenicol and ampicillin.

### Transformation and heterologous expression of killer toxins by *Saccharomyces cerevisiae*

For yeast transformation, the integrative vector containing a killer toxin gene was linearized with the NcoI enzyme by cutting at the *URA3* locus, followed by inactivation of the enzyme at 65°C for 30 min. The product was purified using the Monarch DNA & PCR Cleanup kit (New England Biolabs, Ipswich, MA), following the manufacturer’s recommendations. *S. cerevisiae* CRY1 cells were grown in YPD until an optical density of 1.3 was obtained, and then the cells were washed with sterile water and suspended in 10 mL of TE buffer supplemented with 100 mM LiAc and incubated at 30°C for 30 min under 130 rpm agitation. Cells were recovered by centrifugation at 1,500 × *g* for 5 min at 4°C and suspended in 5 mL of ice-cold 1M sorbitol, followed by centrifugation and suspended in 550 μL of ice-cold 1M sorbitol. For transformation, 80 μL of the suspended cells was added to 5 to 10 μg of the linearized plasmids and transferred to electroporation cuvettes. The cuvettes were incubated on ice for 10 min and pulsed with GenePulser (200 Ω, 1.5 kV, and 25 μF). To recover the transformed cultures, the cells were grown at 30°C for 2 days in complete medium (CM) without uracil (CM: 2.5 g L^−1^ of the drop-out mix without uracil, 1.7 g L^−1^ of yeast nitrogen base YNB, 5 g L^−1^ ammonium sulfate, and 20 g L^−1^ dextrose). For killer toxin expression was induced by overnight growth in YPGal (yeast extract 10 g L^−1^, peptone 20 g L^−1^, and galactose 20 g L^−1^) followed by plating on either YPD or YPGal with citrate-phosphate 29.9 g L^−1^, supplemented with 0.003% wt/vol methylene blue and pH adjusted to 4.6 with HCl and 15 g L^−1^ agar seeded with 10^5^ cells mL^−1^ of a susceptible lawn strain. *C. sinolaborantium* and *S. cerevisiae* CRY1 were used as positive and negative controls for killer activity, respectively.

### Detection of killer toxin expression by RT-PCR

An overnight culture of Ksino was grown in YPD and harvested at OD600 0.9–1.2. Speroplasting was performed by incubating the harvested cells at 30°C for 30 min in buffer Y1 (1M sorbitol, 0.1 M EDTA, pH 7.4, B-ME 0.1%, and 0.1% vol/vol zymolase 20T [10 mg/mL]). Spheroplasts were used as input for a Qiagen RNeasy procedure. The RNA quality was checked using a spectrophotometer, and 10 μg of total RNA was treated with 1 μL of DNase I (ThermoFisher Scientific) in the presence of 10 μL of DNase reaction buffer at 37°C for 10 min and then inactivated by heat at 75°C for 10 min. Transcripts encoding Ksino or actin were detected by RT-PCR (Superscript IV) following the manufacturer’s instructions using the primers 5′-GGCCTTGAGATACCCCATCG-3′ and 5′-CCACGTGAGTAACACCGTCA-3′ (for *ACT1*) and 5′-GATCTACGACAGCAGCCTAGA-3′ and 5′-GTGGTGTACATCGTTGACTCC-3′ (for Ksino). Briefly, the RNA template is denatured with a reverse primer mix at 65°C for 5 min and incubated on ice for at least 1 min. The reverse transcription reaction mix was added to the denatured RNA and incubated for 10 min at 55°C and then inactivated at 80°C for 10 min. A 20 μL PCR was performed with the cycling instructions as follows (98°C for 30 s, then 30 cycles of 98°C for 10 s, 60°C for 30 s, 72°C for 10 s followed by 1 cycle of 72°C for 5 min). PCR products were analyzed by agarose gel electrophoresis.

## Supplementary Material

Legends for Files S1 and S2.

Tables S1 to S15.

File S1 Killer assay data.

File S2 PDB coordinates.

Figures S1 to S9.

The following material is available online.

**File S1 (AEM02246-25-S0001.csv).** Killer assay data.

**File S2 (AEM02246-25-S0002.txt).** PDB coordinates.

**Supplemental figures (AEM02246-25-S0003.pdf). **Figures S1 to S9.

**Supplemental legends (AEM02246-25-S0004.docx).** Legends for Files S1 and S2.

**Supplemental tables (AEM02246-25-S0005.pdf).** Tables S1 to S15.

## Data Availability

Data supporting the results in the paper are available in thesSupplemental material. The GenBank accession number for the open reading frames of the Ksino killer toxin of *Candida sinolaborantium* LESF 1467 is PP790515. The sequenced genome of *Candida sinolaborantium* can be accessed through BioProject PRJNA916362, BioSample SAMN32422847, and Assembly JAQRFW000000000. The raw sequence reads are also available through the NCBI Sequence Read Archive (SRR23081998). Gene prediction and annotation are provided in https://figshare.com/s/1f31cd262fc9de969d38 (10.6084/m9.figshare.25895278).

## IMPORTANCE

Attine ants perform essential ecosystem services through the harvesting of substrates for fungiculture. The cultured fungi are a food source for attine ants. Characterizing antifungal toxin-producing yeasts (killer yeasts) is vital to understanding how they might protect gardens from invasion by unwanted fungal species. This study also describes a new toxin named Ksino from the yeast *Candida sinolaborantium*, a member of a new group of toxins found across many different species of fungi. This work supports the role of killer yeasts in the ecology of fungicultures and competition between fungi. The observed high prevalence of killer yeasts in fungal gardens also enables the discovery of novel antifungal molecules with the potential to be applied against disease-causing fungi.
