# Supplementary material for: Novel killer yeasts and toxins from the gardens of fungus-growing ants
Source: Appl Environ Microbiol. 2026 Jan 21;92(2):e02246-25. doi: 10.1128/aem.02246-25 (PMC12875307; doi:10.1128/aem.02246-25)
Supplement: Supplemental figures — Figures S1 to S9. [file aem.02246-25-s0003.pdf]

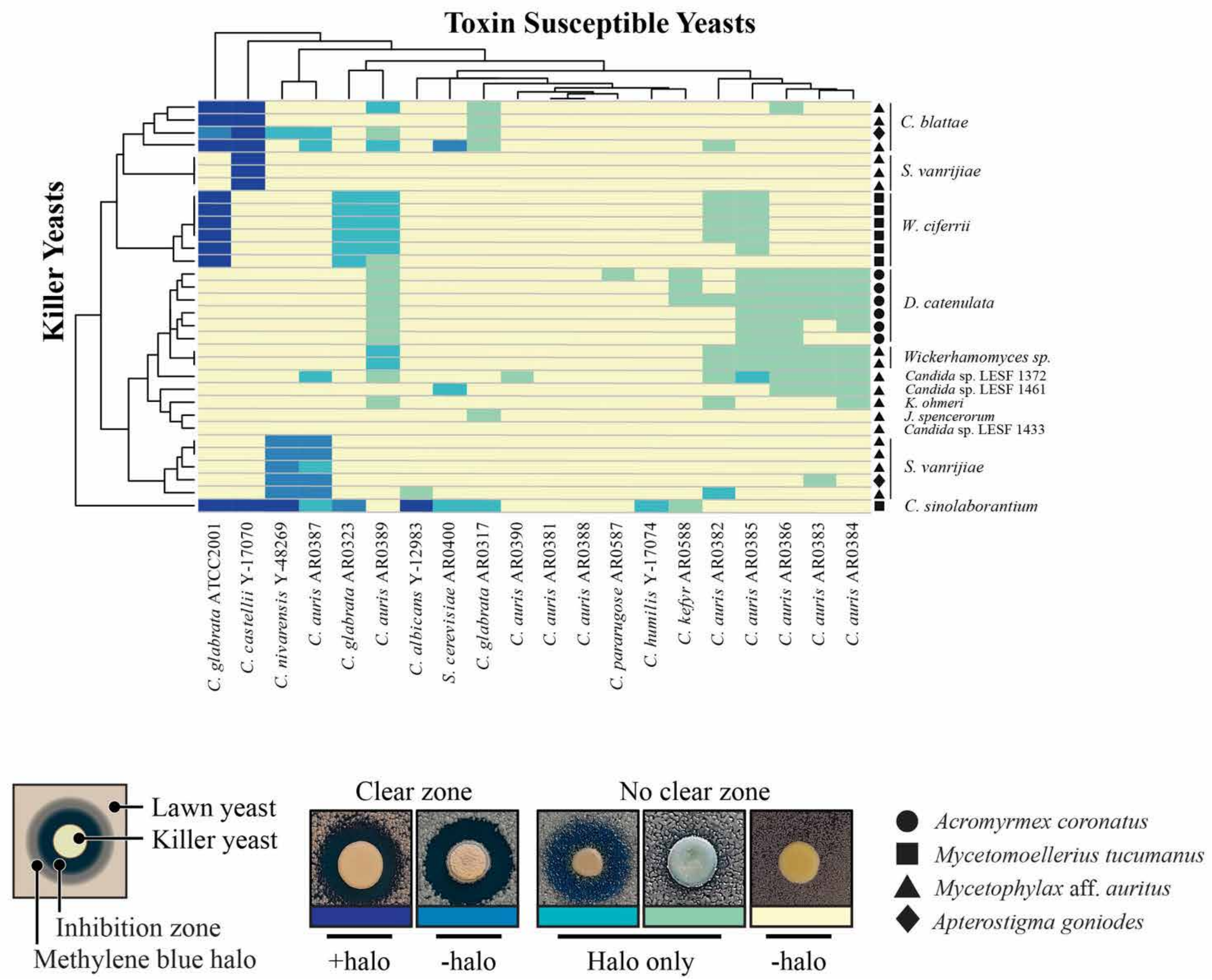

Figure S1. Killer yeasts from attine ant gardens can inhibit the growth of opportunistic human pathogens. A heatmap showing interactions between killer yeasts associated with attine ants and opportunistic human pathogens. Origins of the ant-associated killer yeasts; *Acromyrmex coronatus* (circle); *Mycetomoellerius tucumanus* (square); *Mycetophylax aff. auritus* (triangle), *Apterostigma goniodes* (diamond). Killer toxin activity was qualitatively assessed based on the presence and size of growth inhibition zones and/or methylene blue staining around killer yeasts as diagrammed. Darker colors on the heatmap represent a more prominent killer phenotype, with yellow indicating no observable killer phenotype. Clusters on the dendrograms connecting individual killers or susceptible yeasts indicate similar susceptibilities to killer toxins or antifungal activities. Darker colors on the cluster diagram represent a more prominent killer phenotype, with yellow indicating no observable killer phenotype.

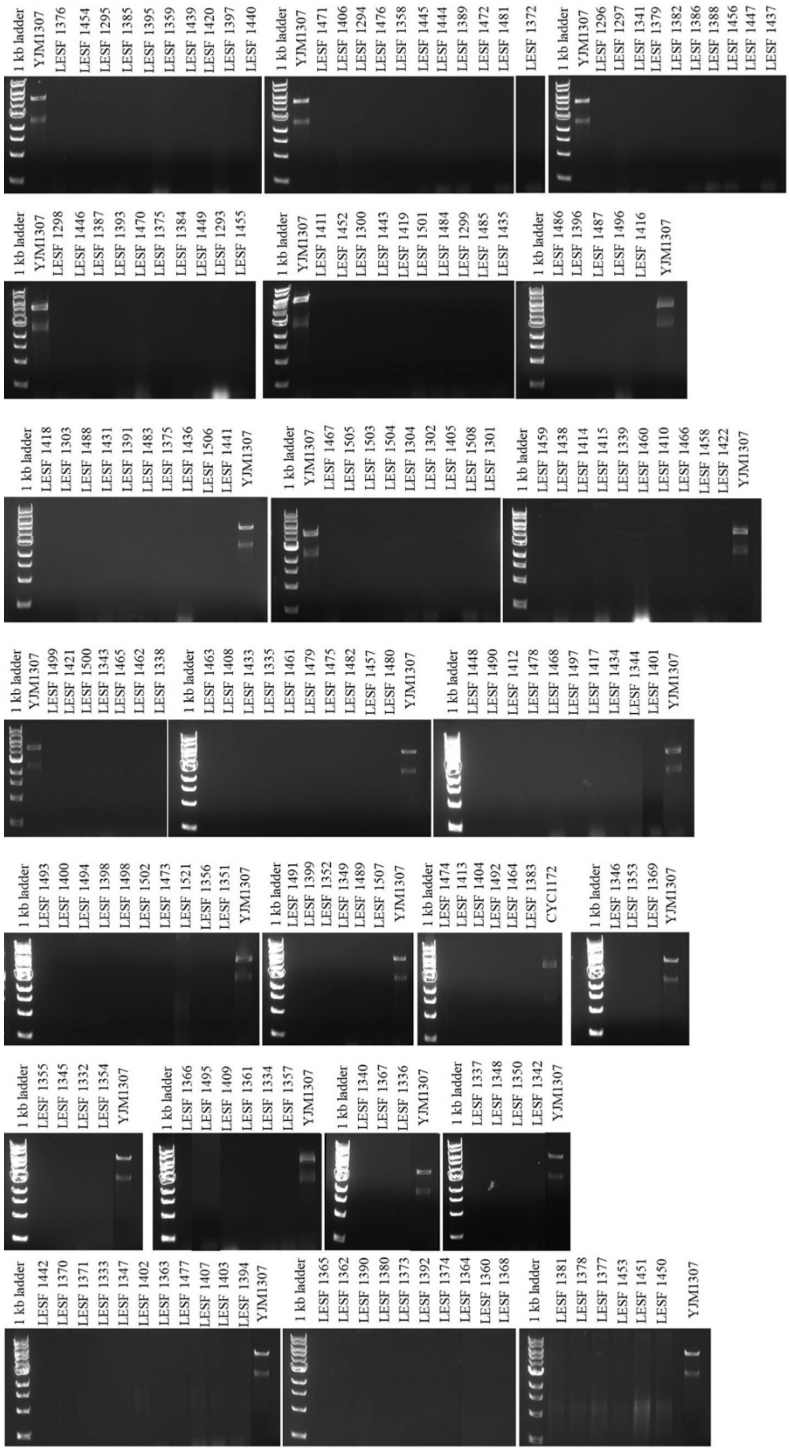

Figure S2. Extraction of dsRNAs using cellulose chromatography from yeasts associated with attine ant fungicultures. *S. cerevisiae* YJM1307 was used as a positive control for dsRNA extraction. Products were visualized by ethidium bromide agarose gel electrophoresis.

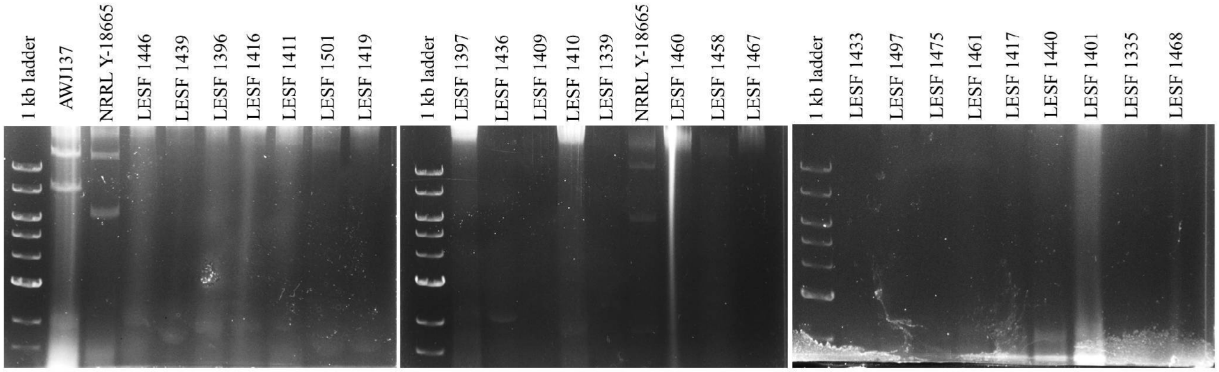

Figure S3. Extraction of dsDNA plasmids from yeasts associated with attine ant fungicultures. *Kluyveromyces lactis* AWJ137 and *Pichia acaciae* NRRL Y-18665 were used as a positive control for plasmid extraction. Products were visualized by ethidium bromide agarose gel electrophoresis.

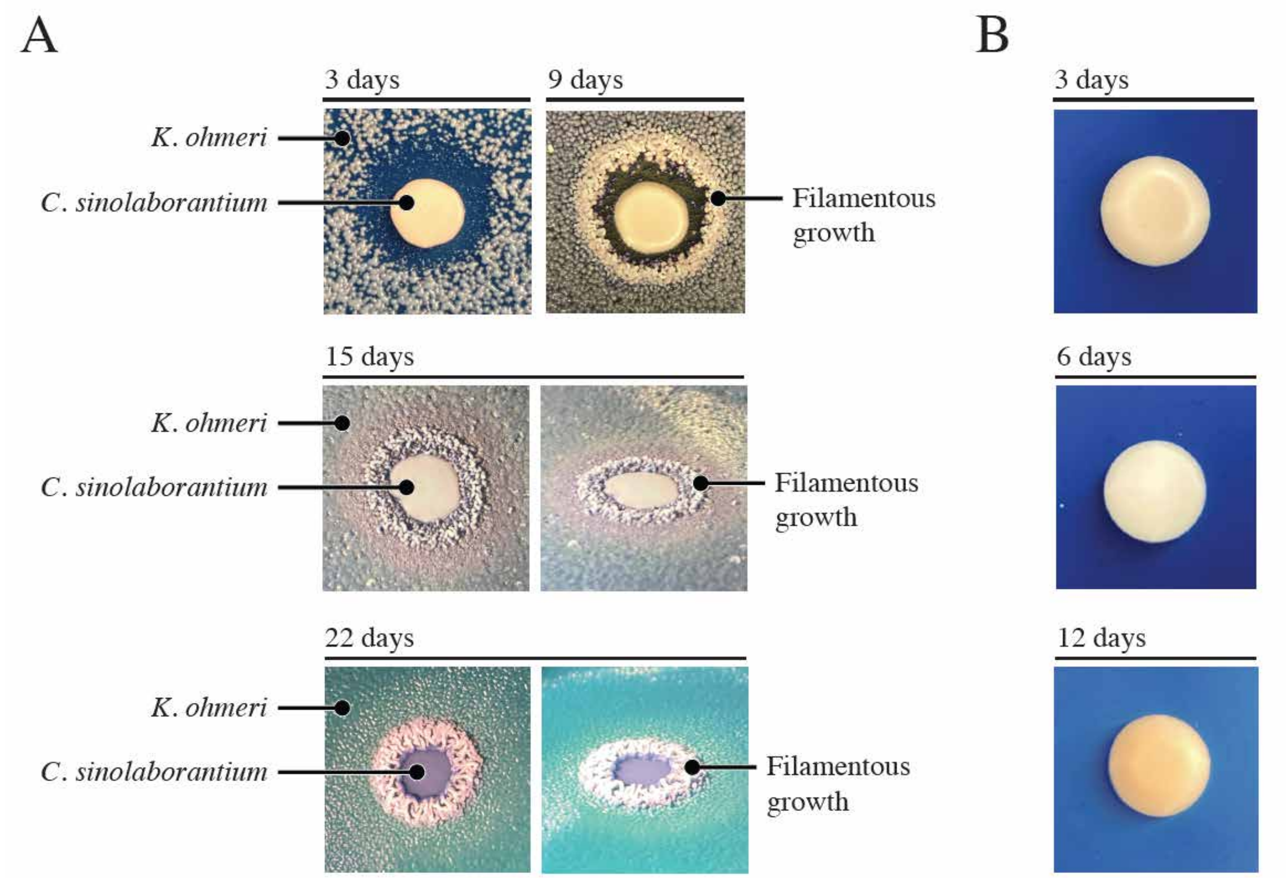

Figure S4. Elongated growth of *K. ohmeri* after coculture with the killer yeast *C. sinolaborantium*. A. Representative images showing the interaction between *K. ohmeri* and the killer yeast *C. sinolaborantium* on YPD agar. After extended incubation times at ~22°C (15 - 22 days), vertical growth from the surface of the agar was observed. B. Elongated growth phenotypes are not observed when *C. sinolaborantium* is grown in isolation on agar.

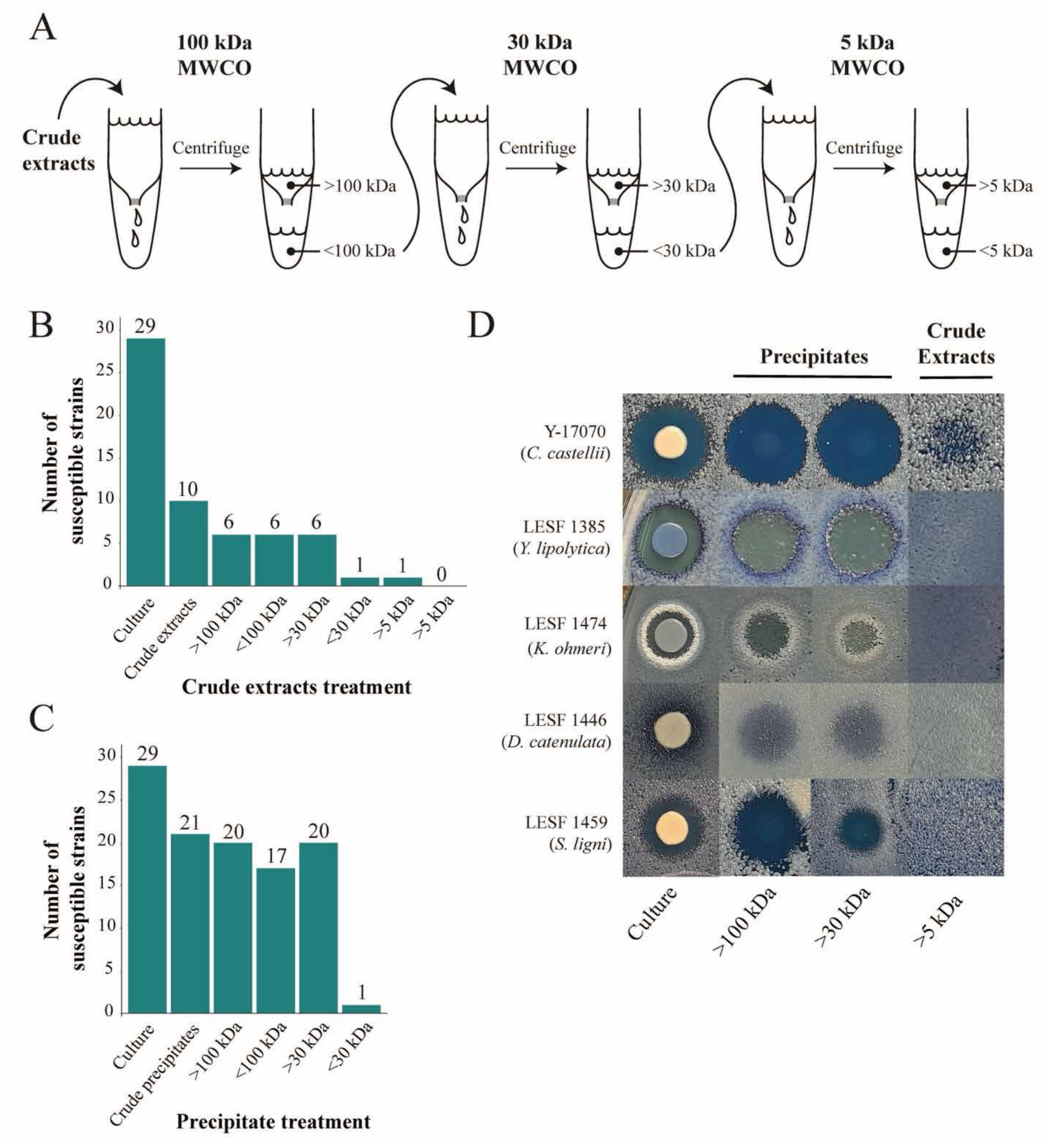

Figure S5. Antifungal activities exhibited by fractionated media after growth of *Candida sinolaborantium*. A. The workflow demonstrates the ultrafiltration of YPD-spent growth media to obtain fractions of antifungal molecules. Crude extracts were processed by ultrafiltration with molecular weight cut-off (MWCO) filters of 100 kDa, 30 kDa, and 5 kDa. The resulting protein preparations were used to challenge susceptible yeasts before (>) and after (<) ultrafiltration. Fractions were used B. before or C. after ethanol-precipitation D. Phenotypic examples of antifungal activity detectable in different fractions against representative susceptible yeast species

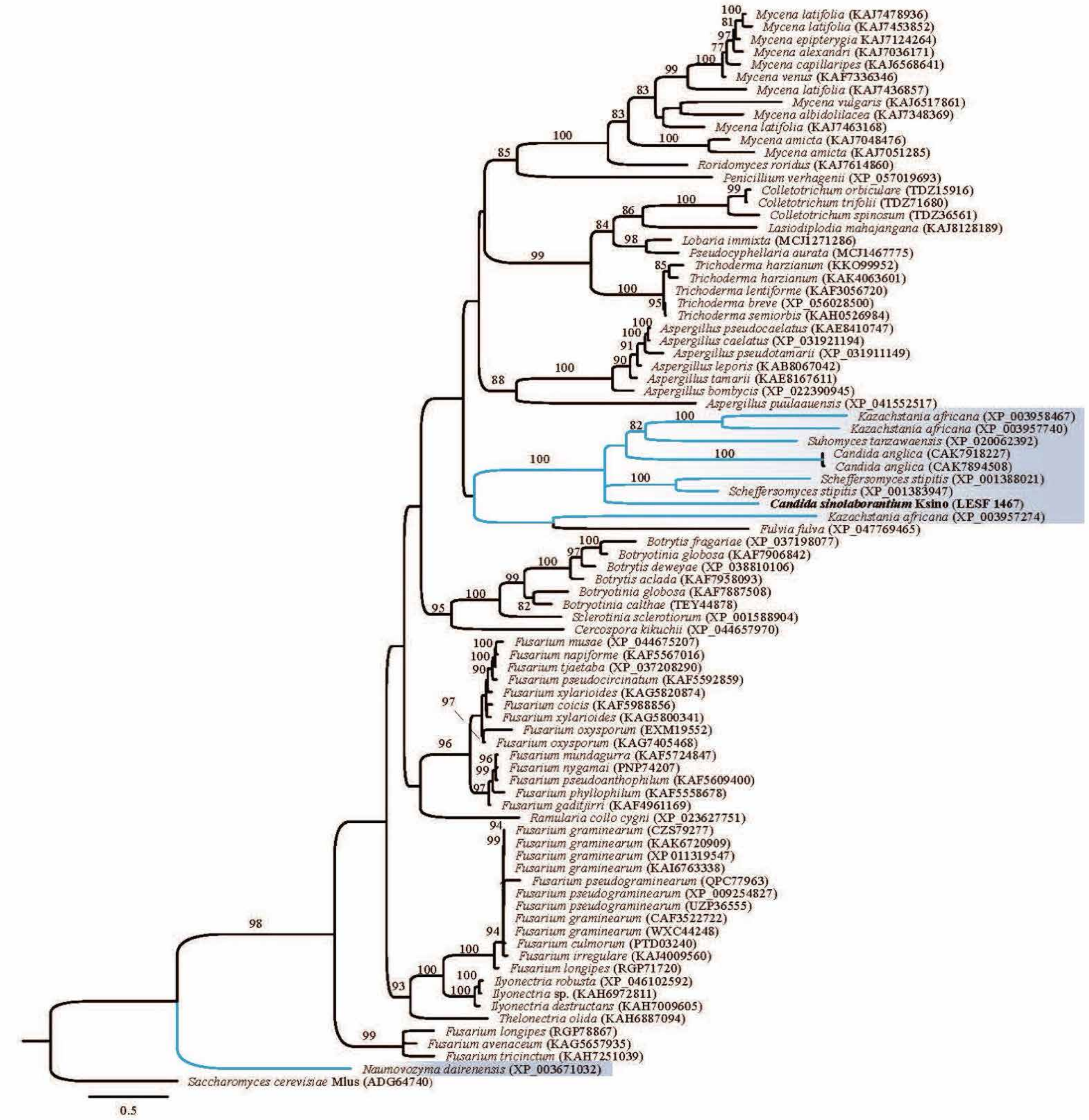

Figure S6. Phylogeny of Ksino homologs identified in diverse fungi. The Ksino-encoding yeast *C. sinolaborantium* is highlighted in bold, and Saccharomycotina in blue. The tree was inferred with maximum likelihood criteria, and numbers on branches are ultrafast bootstrap support values (only values higher than 70 are shown). The dsRNA-encoded killer toxin Klus is represented as an outgroup.

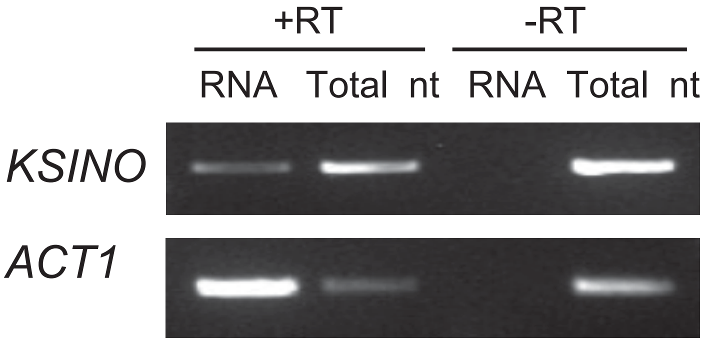

Figure S7. Detection of Ksino expression by RT-PCR. RNA and total nucleic acids (Total nuc.) were extracted from *C. sinolaborantium* grown under laboratory conditions. Primers specific to actin or Ksino were used for RT-PCR and products were visualized by ethidium bromide agarose gel electrophoresis.

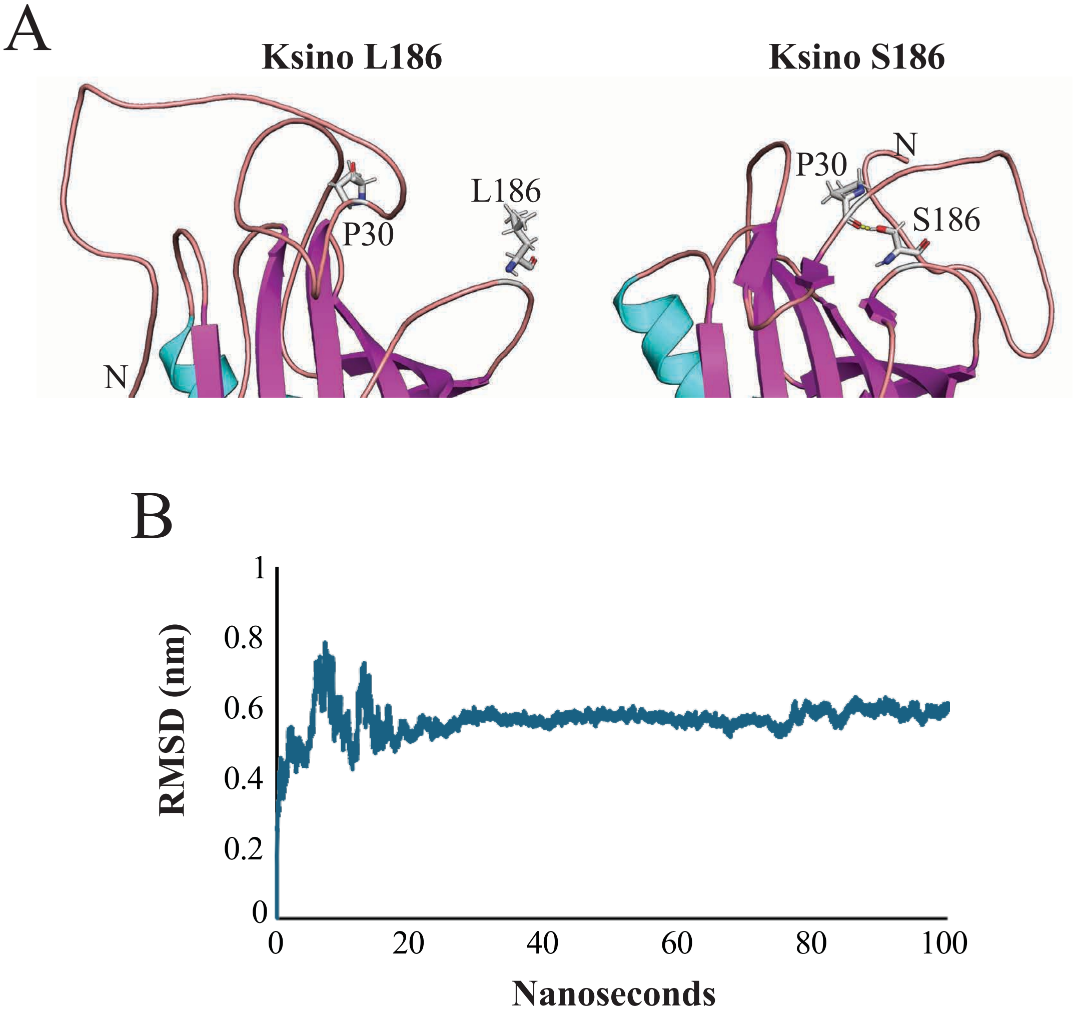

Figure S8. Computational predictions of protein stability with leucine and serine at position 186 in Ksino. A. AlphaFold2 models of Ksino wild type S186 and mutant L186 after 100 ns of molecular dynamics simulation. Residues 186 and P30 are shown as sticks. P30 forms a hydrogen bond (1.6 Å) with the hydroxyl of S186 but is too distant to create this bond in L186. FoldX 5.0 analysis revealed a ΔΔGFolding of 1.0 kcal/mol for L186S. B. 100 ns MD simulation of Ksino L186 AlphaFold2 model. Unlike wild type and Klus, the RMSD stabilizes around 0.55 nm.

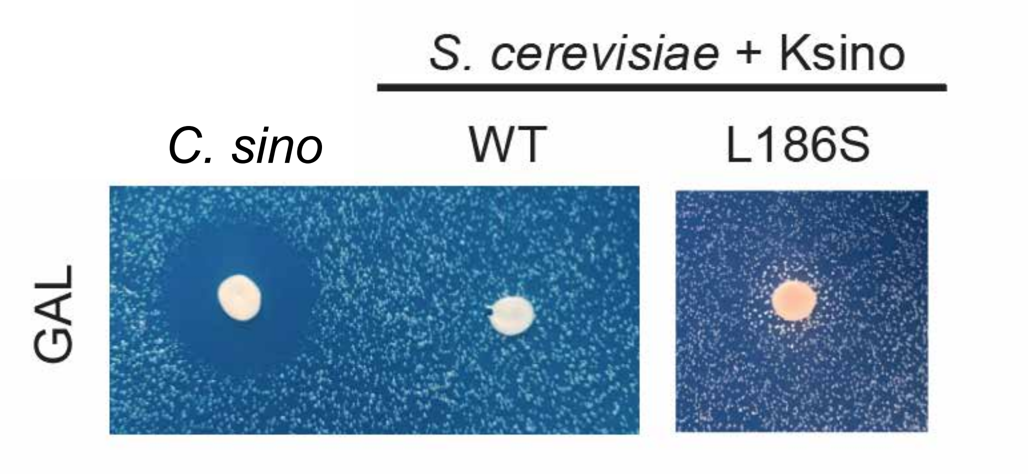

Figure S9. Recombinant Ksino does not inhibit the growth of *K. ohmeri*. Recombinant expression of Ksino (L186S) by *S. cerevisiae* using galactose induction with a lawn of *K. ohmeri* (pH 4.5). The killer phenotype of *C. sinolaborantium* (*C. sino*) was used as a positive control.
