# Supplementary material for: Novel killer yeasts and toxins from the gardens of fungus-growing ants
Source: Appl Environ Microbiol. 2026 Jan 21;92(2):e02246-25. doi: 10.1128/aem.02246-25 (PMC12875307; doi:10.1128/aem.02246-25)
Supplement: Supplemental legends — Legends for Files S1 and S2. [file aem.02246-25-s0004.docx]

File S1 - All killer assay data showing strain names of killer yeasts and potentially susceptible strains of yeast. The severity of growth inhibition is scored from 0-4, with 0 being no inhibition of growth.

File S2 – Coordinates for the structural predictions of Ksino and Klus.
