# Supplementary material for: Novel killer yeasts and toxins from the gardens of fungus-growing ants
Source: Appl Environ Microbiol. 2026 Jan 21;92(2):e02246-25. doi: 10.1128/aem.02246-25 (PMC12875307; doi:10.1128/aem.02246-25)
Supplement: Supplemental tables — Tables S1 to S15. [file aem.02246-25-s0005.pdf]

Table S1. Strains and species of yeasts used in the current study.

Strain	Species	Family	Order	Ant source	Nest ID	City (Brazil)
LESF 1481	*Blastobotrys proliferans*	Trichomonascaceae	Dipodascales	*Acromyrmex coronatus*	RB190919-01	Rio Claro-SP
LESF 1442	*Candida albicans*	Debaryomycetaceae	Serinales	*Acromyrmex coronatus*	RB200104-03	Mogi-Guaçu-SP
LESF 1417	*Candida blattae*	Debaryomycetaceae	Serinales	*Mycetophylax* aff. *auritus*	AR210126-05	Itatiaia-RJ
LESF 1440	*Candida blattae*	Debaryomycetaceae	Serinales	*Mycetophylax* aff. *auritus*	AR210126-05	Itatiaia-RJ
LESF 1401	*Candida blattae*	Debaryomycetaceae	Serinales	*Mycetophylax* aff. *auritus*	AR210126-05	Itatiaia-RJ
LESF 1494	*Candida blattae*	Debaryomycetaceae	Serinales	*Apterostigma goniodes*	AR210127-02	Itatiaia-RJ
LESF 1450	*Candida margitis*	Debaryomycetaceae	Serinales	*Acromyrmex coronatus*	RB190909-01	Rio Claro-SP
LESF 1451	*Candida margitis*	Debaryomycetaceae	Serinales	*Acromyrmex coronatus*	RB190909-01	Rio Claro-SP
LESF 1453	*Candida margitis*	Debaryomycetaceae	Serinales	*Acromyrmex coronatus*	RB190909-01	Rio Claro-SP
LESF 1377	*Candida margitis*	Debaryomycetaceae	Serinales	*Acromyrmex coronatus*	RB190909-01	Rio Claro-SP
LESF 1378	*Candida margitis*	Debaryomycetaceae	Serinales	*Acromyrmex coronatus*	RB190909-01	Rio Claro-SP
LESF 1381	*Candida margitis*	Debaryomycetaceae	Serinales	*Acromyrmex coronatus*	RB190909-01	Rio Claro-SP
LESF 1364	*Candida orthopsilosis*	Debaryomycetaceae	Serinales	*Acromyrmex coronatus*	RB191007-01	Rio Claro-SP
LESF 1394	*Candida orthopsilosis*	Debaryomycetaceae	Serinales	*Acromyrmex coronatus*	RB191007-01	Rio Claro-SP
LESF 1402	*Candida orthopsilosis*	Debaryomycetaceae	Serinales	*Acromyrmex coronatus*	RB191007-01	Rio Claro-SP
LESF 1403	*Candida orthopsilosis*	Debaryomycetaceae	Serinales	*Acromyrmex coronatus*	RB191007-01	Rio Claro-SP
LESF 1477	*Candida orthopsilosis*	Debaryomycetaceae	Serinales	*Acromyrmex coronatus*	RB191007-01	Rio Claro-SP
LESF 1363	*Candida orthopsilosis*	Debaryomycetaceae	Serinales	*Acromyrmex coronatus*	RB191007-01	Rio Claro-SP
LESF 1407	*Candida orthopsilosis*	Debaryomycetaceae	Serinales	*Acromyrmex coronatus*	RB191007-01	Rio Claro-SP
LESF 1373	*Candida orthopsilosis*	Debaryomycetaceae	Serinales	*Acromyrmex coronatus*	RB191007-01	Rio Claro-SP
LESF 1392	*Candida orthopsilosis*	Debaryomycetaceae	Serinales	*Acromyrmex coronatus*	RB191007-01	Rio Claro-SP
LESF 1374	*Candida orthopsilosis*	Debaryomycetaceae	Serinales	*Acromyrmex coronatus*	RB191007-01	Rio Claro-SP
LESF 1380	*Candida orthopsilosis*	Debaryomycetaceae	Serinales	*Acromyrmex coronatus*	RB191007-01	Rio Claro-SP
LESF 1390	*Candida orthopsilosis*	Debaryomycetaceae	Serinales	*Acromyrmex coronatus*	RB191007-01	Rio Claro-SP
LESF 1371	*Candida orthopsilosis*	Debaryomycetaceae	Serinales	*Acromyrmex coronatus*	RB191007-01	Rio Claro-SP

LESF 1370	*Candida orthopsilosis*	Debaryomycetaceae	Serinales	*Acromyrmex coronatus*	RB191007-01	Rio Claro-SP
LESF 1347	*Candida parapsilosis*	Debaryomycetaceae	Serinales	*Mycocepurus goeldii*	RB210430-03	Anhembi-SP
LESF 1333	*Candida parapsilosis*	Debaryomycetaceae	Serinales	*Mycocepurus goeldii*	RB210501-02	Anhembi-SP
LESF 1384	*Candida railenensis*	Debaryomycetaceae	Serinales	*Acromyrmex coronatus*	RB200104-02	Mogi-Guaçu-SP
LESF 1386	*Candida railenensis*	Debaryomycetaceae	Serinales	*Acromyrmex coronatus*	RB200104-02	Mogi-Guaçu-SP
LESF 1387	*Candida railenensis*	Debaryomycetaceae	Serinales	*Acromyrmex coronatus*	RB200104-02	Mogi-Guaçu-SP
LESF 1456	*Candida railenensis*	Debaryomycetaceae	Serinales	*Acromyrmex coronatus*	RB200104-02	Mogi-Guaçu-SP
LESF 1388	*Candida railenensis*	Debaryomycetaceae	Serinales	*Acromyrmex coronatus*	RB200104-02	Mogi-Guaçu-SP
LESF 1455	*Candida railenensis*	Debaryomycetaceae	Serinales	*Acromyrmex coronatus*	RB200104-02	Mogi-Guaçu-SP
LESF 1393	*Candida railenensis*	Debaryomycetaceae	Serinales	*Acromyrmex coronatus*	RB200104-02	Mogi-Guaçu-SP
LESF 1341	*Candida railenensis*	Debaryomycetaceae	Serinales	*Acromyrmex coronatus*	RB200104-02	Mogi-Guaçu-SP
LESF 1437	*Candida railenensis*	Debaryomycetaceae	Serinales	*Acromyrmex coronatus*	RB200104-03	Mogi-Guaçu-SP
LESF 1449	*Candida railenensis*	Debaryomycetaceae	Serinales	*Acromyrmex coronatus*	RB200104-03	Mogi-Guaçu-SP
LESF 1349	*Candida railenensis*	Debaryomycetaceae	Serinales	*Mycocepurus goeldii*	RB210501-06	Anhembi-SP
LESF 1354	*Candida railenensis*	Debaryomycetaceae	Serinales	*Mycocepurus goeldii*	RB210501-06	Anhembi-SP
LESF 1467	*Candida sinolaborantium*	Debaryomycetaceae	Serinales	*Mycetophylax* aff. *auritus*	AR210126-03	Itatiaia-RJ
LESF 1395	*Candida* sp. (closest to *C. intermedia*)	Debaryomycetaceae	Serinales	*Acromyrmex coronatus*	RB191007-01	Rio Claro-SP
LESF 1359	*Candida* sp. (closest to *C. intermedia*)	Debaryomycetaceae	Serinales	*Acromyrmex coronatus*	RB191007-01	Rio Claro-SP
LESF 1433	*Candida* sp. (closest to *C. vrieseae*)	Debaryomycetaceae	Serinales	*Mycetophylax* aff. *auritus*	AR210126-04	Itatiaia-RJ
LESF 1461	*Candida* sp. (closest to *C. temnochilae*)	Debaryomycetaceae	Serinales	*Mycetophylax* aff. *auritus*	AR210126-05	Itatiaia-RJ
LESF 1372	*Candida* sp. (closest to *C. temnochilae*)	Debaryomycetaceae	Serinales	*Mycetophylax* aff. *auritus*	PK210128-01	Itatiaia-RJ
LESF 1413	*Candida* sp. (closest to *C. auris*)	Debaryomycetaceae	Serinales	*Mycetophylax* aff. *auritus*	PK210128-01	Itatiaia-RJ
LESF 1493	*Candida* sp. (closest to *C. vrieseae*)	Debaryomycetaceae	Serinales	*Apterostigma goniodes*	AR210127-02	Itatiaia-RJ

LESF 1438	*Cyberlindnera* sp.	Phaffomycetaceae	Phaffomycetales	*Mycetophylax* aff. *auritus*	AR210126-02	Itatiaia-RJ
LESF 1405	*Debaryomyces hansenii*	Debaryomycetaceae	Serinales	*Acromyrmex coronatus*	RB191007-01	Rio Claro-SP
LESF 1446	*Diutina catenulata*	Debaryomycetaceae	Serinales	*Acromyrmex coronatus*	RB200104-03	Mogi-Guaçu-SP
LESF 1439	*Diutina catenulata*	Debaryomycetaceae	Serinales	*Acromyrmex coronatus*	RB200104-03	Mogi-Guaçu-SP
LESF 1396	*Diutina catenulata*	Debaryomycetaceae	Serinales	*Acromyrmex coronatus*	RB200104-03	Mogi-Guaçu-SP
LESF 1416	*Diutina catenulata*	Debaryomycetaceae	Serinales	*Acromyrmex coronatus*	RB200104-03	Mogi-Guaçu-SP
LESF 1501	*Diutina catenulata*	Debaryomycetaceae	Serinales	*Acromyrmex coronatus*	RB200104-03	Mogi-Guaçu-SP
LESF 1419	*Diutina catenulata*	Debaryomycetaceae	Serinales	*Acromyrmex coronatus*	RB200104-03	Mogi-Guaçu-SP
LESF 1345	*Grigorovia* sp.	Saccharomycetaceae	Saccharomycetales	*Mycocepurus goeldii*	RB210501-06	Anhembi-SP
LESF 1470	*Hanseniaspora opuntiae*	Saccharomycodaceae	Saccharomycodales	*Acromyrmex coronatus*	RB200104-03	Mogi-Guaçu-SP
LESF 1488	*Hanseniaspora opuntiae*	Saccharomycodaceae	Saccharomycodales	*Acromyrmex coronatus*	RB200104-03	Mogi-Guaçu-SP
LESF 1484	*Hanseniaspora opuntiae*	Saccharomycodaceae	Saccharomycodales	*Acromyrmex coronatus*	RB200104-03	Mogi-Guaçu-SP
LESF 1297	*Hanseniaspora thailandica*	Saccharomycodaceae	Saccharomycodales	*Acromyrmex coronatus*	RB200104-02	Mogi-Guaçu-SP
LESF 1298	*Hanseniaspora thailandica*	Saccharomycodaceae	Saccharomycodales	*Acromyrmex coronatus*	RB200104-02	Mogi-Guaçu-SP
LESF 1483	*Hanseniaspora thailandica*	Saccharomycodaceae	Saccharomycodales	*Acromyrmex coronatus*	RB200104-03	Mogi-Guaçu-SP
LESF 1435	*Hanseniaspora thailandica*	Saccharomycodaceae	Saccharomycodales	*Acromyrmex coronatus*	RB200104-03	Mogi-Guaçu-SP
LESF 1486	*Hanseniaspora thailandica*	Saccharomycodaceae	Saccharomycodales	*Acromyrmex coronatus*	RB200104-04	Mogi-Guaçu-SP
LESF 1503	*Hyphopichia burtonii*	Debaryomycetaceae	Serinales	*Mycetomoellerius tucumanus*	AR201207-02	Rio Claro-SP
LESF 1505	*Hyphopichia burtonii*	Debaryomycetaceae	Serinales	*Mycetomoellerius tucumanus*	AR201207-02	Rio Claro-SP
LESF 1504	*Hyphopichia burtonii*	Debaryomycetaceae	Serinales	*Mycetomoellerius tucumanus*	AR201207-02	Rio Claro-SP
LESF 1499	*Hyphopichia burtonii*	Debaryomycetaceae	Serinales	*Mycetomoellerius tucumanus*	RB201207-01	Rio Claro-SP
LESF 1344	*Hyphopichia* sp.	Debaryomycetaceae	Serinales	*Mycetophylax* aff. *auritus*	AR210126-04	Itatiaia-RJ
LESF 1468	*Jamesozyma spencerorum*	Saccharomycetaceae	Saccharomycetales	*Mycetophylax* aff. *auritus*	RB210126-02	Itatiaia-RJ
LESF 1474	*Kodamaea ohmeri*	Debaryomycetaceae	Serinales	*Mycetophylax* aff. *auritus*	PK210128-01	Itatiaia-RJ

LESF 1421	*Kurtzmaniella quercitrusa*	Debaryomycetaceae	Serinales	*Mycetophylax* aff. *auritus*	AR210126-01	Itatiaia-RJ
LESF 1489	*Kurtzmaniella quercitrusa*	Debaryomycetaceae	Serinales	*Apterostigma goniodes*	RB210126-03	Itatiaia-RJ
LESF 1398	*Kurtzmaniella quercitrusa*	Debaryomycetaceae	Serinales	*Apterostigma goniodes*	RB210126-03	Itatiaia-RJ
LESF 1400	*Kurtzmaniella quercitrusa*	Debaryomycetaceae	Serinales	*Apterostigma goniodes*	AR210127-02	Itatiaia-RJ
LESF 1498	*Kurtzmaniella quercitrusa*	Debaryomycetaceae	Serinales	*Apterostigma goniodes*	AR210127-02	Itatiaia-RJ
LESF 1399	*Kurtzmaniella quercitrusa*	Debaryomycetaceae	Serinales	*Apterostigma goniodes*	AR210127-02	Itatiaia-RJ
LESF 1473	*Kurtzmaniella* sp.	Debaryomycetaceae	Serinales	*Mycetophylax* aff. *auritus*	PK210126-03	Itatiaia-RJ
LESF 1491	*Kurtzmaniella* sp.	Debaryomycetaceae	Serinales	*Mycetophylax* aff. *auritus*	PK210128-01	Itatiaia-RJ
LESF 1500	*Limtongozyma cylindracea*	Debaryomycetaceae	Serinales	*Mycetophylax* aff. *auritus*	AR210126-02	Itatiaia-RJ
LESF 1404	*Limtongozyma* sp.	Debaryomycetaceae	Serinales	*Mycetophylax* aff. *auritus*	PK210126-03	Itatiaia-RJ
LESF 1492	*Limtongozyma* sp.	Debaryomycetaceae	Serinales	*Mycetophylax* aff. *auritus*	PK210128-01	Itatiaia-RJ
LESF 1368	*Lodderomyces elongisporus*	Debaryomycetaceae	Serinales	*Acromyrmex coronatus*	RB191007-01	Rio Claro-SP
LESF 1360	*Lodderomyces elongisporus*	Debaryomycetaceae	Serinales	*Acromyrmex coronatus*	RB191007-01	Rio Claro-SP
LESF 1362	*Lodderomyces elongisporus*	Debaryomycetaceae	Serinales	*Acromyrmex coronatus*	RB191007-01	Rio Claro-SP
LESF 1365	*Lodderomyces elongisporus*	Debaryomycetaceae	Serinales	*Acromyrmex coronatus*	RB191007-01	Rio Claro-SP
LESF 1422	*Metschnikowia koreensis*	Metschnikowiaceae	Serinales	*Mycetomoellerius tucumanus*	AR201207-03	Rio Claro-SP
LESF 1343	*Metschnikowia* sp.	Metschnikowiaceae	Serinales	*Mycetomoellerius tucumanus*	LV201207-01	Rio Claro-SP
LESF 1415	*Metschnikowia* sp.	Metschnikowiaceae	Serinales	*Mycetomoellerius tucumanus*	LV201207-01	Rio Claro-SP
LESF 1383	*Metschnikowia* sp.	Metschnikowiaceae	Serinales	*Mycetophylax* aff. *auritus*	PK210128-01	Itatiaia-RJ
LESF 1355	*Metschnikowia* sp.	Metschnikowiaceae	Serinales	*Mycocepurus goeldii*	RB210501-05	Anhembi-SP
LESF 1332	*Metschnikowia* sp.	Metschnikowiaceae	Serinales	*Mycocepurus goeldii*	RB210501-05	Anhembi-SP

LESF 1375	*Meyerozyma caribbica*	Debaryomycetaceae	Serinales	*Acromyrmex coronatus*	RB191007-01	Rio Claro-SP
LESF 1382	*Meyerozyma caribbica*	Debaryomycetaceae	Serinales	*Acromyrmex coronatus*	RB191007-01	Rio Claro-SP
LESF 1443	*Meyerozyma caribbica*	Debaryomycetaceae	Serinales	*Acromyrmex coronatus*	RB200104-03	Mogi-Guaçu-SP
LESF 1452	*Meyerozyma caribbica*	Debaryomycetaceae	Serinales	*Acromyrmex coronatus*	RB200104-03	Mogi-Guaçu-SP
LESF 1389	*Meyerozyma carpophila*	Debaryomycetaceae	Serinales	*Acromyrmex coronatus*	RB191007-01	Rio Claro-SP
LESF 1406	*Meyerozyma guilliermondii*	Debaryomycetaceae	Serinales	*Acromyrmex coronatus*	RB191007-01	Rio Claro-SP
LESF 1444	*Meyerozyma guilliermondii*	Debaryomycetaceae	Serinales	*Acromyrmex coronatus*	RB191007-01	Rio Claro-SP
LESF 1379	*Meyerozyma guilliermondii*	Debaryomycetaceae	Serinales	*Acromyrmex coronatus*	RB191007-01	Rio Claro-SP
LESF 1411	*Pichia kudriavzevii*	Pichiaceae	Pichiales	*Acromyrmex coronatus*	RB200104-03	Mogi-Guaçu-SP
LESF 1391	*Priceomyces* sp.	Debaryomycetaceae	Serinales	*Acromyrmex coronatus*	RB200507-03	Rio Claro-SP
LESF 1445	Saccharomycetales sp.	Debaryomycetaceae	Serinales	*Acromyrmex coronatus*	RB190919-01	Rio Claro-SP
LESF 1457	*Scheffersomyces* sp.	Debaryomycetaceae	Serinales	*Mycetophylax* aff. *auritus*	AR210126-05	Itatiaia-RJ
LESF 1448	*Scheffersomyces* sp.	Debaryomycetaceae	Serinales	*Mycetophylax* aff. *auritus*	RB210126-02	Itatiaia-RJ
LESF 1408	*Scheffersomyces* sp.	Debaryomycetaceae	Serinales	*Mycetophylax* aff. *auritus*	PK210126-03	Itatiaia-RJ
LESF 1464	*Scheffersomyces* sp.	Debaryomycetaceae	Serinales	*Mycetophylax* aff. *auritus*	PK210126-03	Itatiaia-RJ
LESF 1497	*Schwanniomyces vanrijiae*	Debaryomycetaceae	Serinales	*Mycetophylax* aff. *auritus*	AR210126-04	Itatiaia-RJ
LESF 1475	*Schwanniomyces vanrijiae*	Debaryomycetaceae	Serinales	*Mycetophylax* aff. *auritus*	AR210126-05	Itatiaia-RJ
LESF 1335	*Schwanniomyces vanrijiae*	Debaryomycetaceae	Serinales	*Mycetophylax* aff. *auritus*	RB210126-02	Itatiaia-RJ
LESF 1482	*Schwanniomyces vanrijiae*	Debaryomycetaceae	Serinales	*Mycetophylax* aff. *auritus*	RB210126-02	Itatiaia-RJ
LESF 1480	*Schwanniomyces vanrijiae*	Debaryomycetaceae	Serinales	*Mycetophylax* aff. *auritus*	RB210126-02	Itatiaia-RJ
LESF 1478	*Schwanniomyces vanrijiae*	Debaryomycetaceae	Serinales	*Mycetophylax* aff. *auritus*	RB210126-02	Itatiaia-RJ
LESF 1479	*Schwanniomyces vanrijiae*	Debaryomycetaceae	Serinales	*Mycetophylax* aff. *auritus*	RB210126-02	Itatiaia-RJ

LESF 1521	*Schwanniomyces vanrijiae*	Debaryomycetaceae	Serinales	*Apterostigma goniodes*	AR210127-02	Itatiaia-RJ
LESF 1472	*Starmerella bombicola*	Trichomonascaceae	Dipodascales	*Acromyrmex coronatus*	RB190909-01	Rio Claro-SP
LESF 1376	*Starmerella bombicola*	Trichomonascaceae	Dipodascales	*Acromyrmex coronatus*	RB190909-01	Rio Claro-SP
LESF 1476	*Starmerella bombicola*	Trichomonascaceae	Dipodascales	*Acromyrmex coronatus*	RB190909-01	Rio Claro-SP
LESF 1471	*Starmerella bombicola*	Trichomonascaceae	Dipodascales	*Acromyrmex coronatus*	RB190909-01	Rio Claro-SP
LESF 1487	*Starmerella etchellsii*	Trichomonascaceae	Dipodascales	*Acromyrmex coronatus*	RB200507-01	Rio Claro-SP
LESF 1299	*Starmerella etchellsii*	Trichomonascaceae	Dipodascales	*Acromyrmex coronatus*	RB200507-01	Rio Claro-SP
LESF 1300	*Starmerella etchellsii*	Trichomonascaceae	Dipodascales	*Acromyrmex coronatus*	RB200507-01	Rio Claro-SP
LESF 1485	*Starmerella etchellsii*	Trichomonascaceae	Dipodascales	*Acromyrmex coronatus*	RB200507-01	Rio Claro-SP
LESF 1301	*Starmerella etchellsii*	Trichomonascaceae	Dipodascales	*Acromyrmex coronatus*	RB200507-01	Rio Claro-SP
LESF 1304	*Starmerella etchellsii*	Trichomonascaceae	Dipodascales	*Acromyrmex coronatus*	RB200507-01	Rio Claro-SP
LESF 1302	*Starmerella etchellsii*	Trichomonascaceae	Dipodascales	*Acromyrmex coronatus*	RB200507-01	Rio Claro-SP
LESF 1303	*Starmerella etchellsii*	Trichomonascaceae	Dipodascales	*Acromyrmex coronatus*	RB200507-01	Rio Claro-SP
LESF 1508	*Starmerella etchellsii*	Trichomonascaceae	Dipodascales	*Acromyrmex coronatus*	RB200507-01	Rio Claro-SP
LESF 1496	*Starmerella meliponinorum*	Trichomonascaceae	Dipodascales	*Acromyrmex coronatus*	RB200507-01	Rio Claro-SP
LESF 1420	*Starmerella meliponinorum*	Trichomonascaceae	Dipodascales	*Acromyrmex coronatus*	RB200507-01	Rio Claro-SP
LESF 1418	*Starmerella meliponinorum*	Trichomonascaceae	Dipodascales	*Acromyrmex coronatus*	RB200507-01	Rio Claro-SP
LESF 1293	*Starmerella* sp.	Trichomonascaceae	Dipodascales	*Acromyrmex coronatus*	RB190909-01	Rio Claro-SP
LESF 1454	*Starmerella* sp.	Trichomonascaceae	Dipodascales	*Acromyrmex coronatus*	RB190919-01	Rio Claro-SP
LESF 1294	*Starmerella* sp.	Trichomonascaceae	Dipodascales	*Acromyrmex coronatus*	RB190919-01	Rio Claro-SP
LESF 1295	*Starmerella* sp.	Trichomonascaceae	Dipodascales	*Acromyrmex coronatus*	RB190919-01	Rio Claro-SP
LESF 1431	*Starmerella* sp.	Trichomonascaceae	Dipodascales	*Acromyrmex coronatus*	RB200507-01	Rio Claro-SP
LESF 1465	*Sugiyamaella bonitensis*	Trichomonascaceae	Dipodascales	*Mycetophylax* aff. *auritus*	AR210126-01	Itatiaia-RJ
LESF 1366	*Sugiyamaella boreocaroliniensis*	Trichomonascaceae	Dipodascales	*Mycocepurus goeldii*	RB210430-06	Anhembi-SP

LESF 1463	*Sugiyamaella carassensis*	Trichomonascaceae	Dipodascales	*Mycetophylax* aff. *auritus*	AR210126-01	Itatiaia-RJ
LESF 1338	*Sugiyamaella carassensis*	Trichomonascaceae	Dipodascales	*Mycetophylax* aff. *auritus*	AR210126-01	Itatiaia-RJ
LESF 1356	*Sugiyamaella carassensis*	Trichomonascaceae	Dipodascales	*Mycocepurus goeldii*	RB210501-06	Anhembi-SP
LESF 1459	*Sugiyamaella ligni*	Trichomonascaceae	Dipodascales	*Mycetophylax* aff. *auritus*	AR210126-01	Itatiaia-RJ
LESF 1414	*Sugiyamaella ligni*	Trichomonascaceae	Dipodascales	*Mycetophylax* aff. *auritus*	AR210126-01	Itatiaia-RJ
LESF 1462	*Sugiyamaella ligni*	Trichomonascaceae	Dipodascales	*Mycetophylax* aff. *auritus*	AR210126-01	Itatiaia-RJ
LESF 1351	*Sugiyamaella smithiae*	Trichomonascaceae	Dipodascales	*Mycocepurus goeldii*	RB210430-06	Anhembi-SP
LESF 1352	*Sugiyamaella smithiae*	Trichomonascaceae	Dipodascales	*Mycocepurus goeldii*	RB210430-06	Anhembi-SP
LESF 1353	*Sugiyamaella smithiae*	Trichomonascaceae	Dipodascales	*Mycocepurus goeldii*	RB210430-06	Anhembi-SP
LESF 1334	*Sugiyamaella smithiae*	Trichomonascaceae	Dipodascales	*Mycocepurus goeldii*	RB210430-06	Anhembi-SP
LESF 1357	*Sugiyamaella smithiae*	Trichomonascaceae	Dipodascales	*Mycocepurus goeldii*	RB210430-06	Anhembi-SP
LESF 1348	*Sugiyamaella smithiae*	Trichomonascaceae	Dipodascales	*Mycocepurus goeldii*	RB210430-06	Anhembi-SP
LESF 1369	*Sugiyamaella smithiae*	Trichomonascaceae	Dipodascales	*Mycocepurus goeldii*	RB210430-06	Anhembi-SP
LESF 1367	*Sugiyamaella smithiae*	Trichomonascaceae	Dipodascales	*Mycocepurus goeldii*	RB210430-06	Anhembi-SP
LESF 1361	*Sugiyamaella smithiae*	Trichomonascaceae	Dipodascales	*Mycocepurus goeldii*	RB210430-06	Anhembi-SP
LESF 1350	*Sugiyamaella smithiae*	Trichomonascaceae	Dipodascales	*Mycocepurus goeldii*	RB210430-06	Anhembi-SP
LESF 1337	*Sugiyamaella smithiae*	Trichomonascaceae	Dipodascales	*Mycocepurus goeldii*	RB210430-06	Anhembi-SP
LESF 1336	*Sugiyamaella smithiae*	Trichomonascaceae	Dipodascales	*Mycocepurus goeldii*	RB210430-06	Anhembi-SP
LESF 1340	*Sugiyamaella smithiae*	Trichomonascaceae	Dipodascales	*Mycocepurus goeldii*	RB210430-06	Anhembi-SP
LESF 1342	*Sugiyamaella smithiae*	Trichomonascaceae	Dipodascales	*Mycocepurus goeldii*	RB210430-06	Anhembi-SP
LESF 1346	*Sugiyamaella smithiae*	Trichomonascaceae	Dipodascales	*Mycocepurus goeldii*	RB210430-06	Anhembi-SP
LESF 1296	*Wickerhamiella occidentalis*	Trichomonascaceae	Dipodascales	*Acromyrmex coronatus*	RB200104-01	Mogi-Guaçu-SP
LESF 1502	*Wickerhamiella occidentalis*	Trichomonascaceae	Dipodascales	*Mycetophylax* aff. *auritus*	PK210126-03	Itatiaia-RJ
LESF 1506	*Wickerhamiella* sp.	Trichomonascaceae	Dipodascales	*Acromyrmex coronatus*	RB200507-02	Rio Claro-SP

LESF 1441	*Wickerhamiella* sp.	Trichomonascaceae	Dipodascales	*Acromyrmex coronatus*	RB200507-02	Rio Claro-SP
LESF 1495	*Wickerhamiella* sp.	Trichomonascaceae	Dipodascales	*Mycocepurus goeldii*	RB210430-06	Anhembi-SP
LESF 1397	*Wickerhamomyces ciferrii*	Wickerhamomycetaceae	Phaffomycetales	*Mycetomoellerius tucumanus*	AR201207-02	Rio Claro-SP
LESF 1436	*Wickerhamomyces ciferrii*	Wickerhamomycetaceae	Phaffomycetales	*Mycetomoellerius tucumanus*	AR201207-02	Rio Claro-SP
LESF 1409	*Wickerhamomyces ciferrii*	Wickerhamomycetaceae	Phaffomycetales	*Mycetomoellerius tucumanus*	AR201207-02	Rio Claro-SP
LESF 1410	*Wickerhamomyces ciferrii*	Wickerhamomycetaceae	Phaffomycetales	*Mycetomoellerius tucumanus*	AR201207-02	Rio Claro-SP
LESF 1339	*Wickerhamomyces ciferrii*	Wickerhamomycetaceae	Phaffomycetales	*Mycetomoellerius tucumanus*	AR201207-02	Rio Claro-SP
LESF 1466	*Wickerhamomyces ciferrii*	Wickerhamomycetaceae	Phaffomycetales	*Mycetomoellerius tucumanus*	RB201207-01	Rio Claro-SP
LESF 1460	*Wickerhamomyces* sp.	Wickerhamomycetaceae	Phaffomycetales	*Mycetophylax* aff. *auritus*	AR210126-02	Itatiaia-RJ
LESF 1458	*Wickerhamomyces* sp.	Wickerhamomycetaceae	Phaffomycetales	*Mycetophylax* aff. *auritus*	AR210126-02	Itatiaia-RJ
LESF 1412	*Yamadazyma paraaseri*	Debaryomycetaceae	Serinales	*Mycetophylax* aff. *auritus*	AR210126-02	Itatiaia-RJ
LESF 1434	*Yamadazyma* sp.	Debaryomycetaceae	Serinales	*Mycetophylax* aff. *auritus*	AR210126-03	Itatiaia-RJ
LESF 1490	*Yamadazyma* sp.	Debaryomycetaceae	Serinales	*Mycetophylax* aff. *auritus*	AR210126-03	Itatiaia-RJ
LESF 1507	*Yamadazyma* sp.	Debaryomycetaceae	Serinales	*Apterostigma goniodes*	AR210127-02	Itatiaia-RJ
LESF 1385	*Yarrowia lipolytica*	Incertis sedis	Dipodascales	*Acromyrmex coronatus*	RB191007-01	Rio Claro-SP
LESF 1358	*Yarrowia lipolytica*	Incertis sedis	Dipodascales	*Acromyrmex coronatus*	RB191007-01	Rio Claro-SP
LESF 1447	*Yarrowia lipolytica*	Incertis sedis	Dipodascales	*Acromyrmex coronatus*	RB191007-01	Rio Claro-SP

Table S2. Nucleotide sequences used in phylogenetic analysis of yeast species delimitation.

Yeast species	Isolate	GenBank accession
Babjeviella inositovora	NRRL Y-12698	JQ689053
Blastobotrys proliferans	LESF 1481	ON493984
Blastobotrys proliferans	NRRL Y-17577	DQ442684
Candida aaseri	NRRL YB-3897	U45802
Candida africana	UPV-EHU 97135	AM495238
Candida albicans	LESF 1442	ON493939
Candida albicans	NRRL Y-12983	U45776
Candida auris	143	AB375773
Candida blattae	LESF 1417	ON493963
Candida blattae	ATCC MYA-4360	FJ614695
Candida blattariae	NRRL Y-27703	AY640213
Candida dosseyi	NRRL Y-27950	DQ655681
Candida endomychidarum	NRRL Y-27708	AY520330
Candida haemuloni	CBS 5149	KY106490
Candida insectorum	NRRL Y-7787	U45791
Candida intermedia	NRRL Y-981	U44809
Candida loeiensis	NBRC 106731	AB602835
Candida margitis	LESF 1378	ON493906
Candida margitis	NYNU 15857	KU128721
Candida membranifaciens	NRRL Y-2089	U45792
Candida nanaspora	NRRL Y-17679	U70187
Candida orthopsilosis	LESF 1402	ON493923
Candida orthopsilosis	MCO456	DQ213056
Candida oxycetoniae	AS 2.3656	EU402933
Candida parapsilosis	LESF 1347	ON493997

Candida parapsilosis	NRRL Y-12969	U45754
Candida powellii	UWO(PS)99-325.3	AF251554
Candida pseudoaaseri	CBS 11170	JN241689
Candida pseudointermedia	CBS 6918	KY106703
Candida quercuum	NRRL Y-12942	EF550292
Candida railenensis	LESF 1437	ON493931
Candida railenensis	NRRL Y-17762	U45800
Candida ruelliae	MTCC 7739	AM262326
Candida sinolaborantium	LESF 1467	ON493969
Candida sinolaborantium	ATCC MYA-4337	FJ614676
Candida sp.	BG01-7-23-017A-1-2	AY242310
Candida sp.	NRRL Y-27166	EU011603
Candida sp.	NRRL Y-12764	EU011605
Candida sp.	05-7-186T	FJ537069
Candida sp.	DMKU-WBL1-3	MH744637
Candida sp. (closest to *C. intermedia*)	LESF 1395	ON493958
Candida sp. (closest to *C. vrieseae*)	LESF 1433	ON493964
Candida sp. (closest to *C. auris*)	LESF 1413	ON493965
Candida sp. (closest to *C. temnochilae*)	LESF 1461	ON493970
Candida sp. (closest to *C. vrieseae*)	LESF 1493	ON493972
Candida temnochilae	CBS 9938	AY242344
Candida vrieseae	BI146	EU200785
Cyberlindnera fabianii	NRRL Y-1871	EF550321
Cyberlindnera sp.	LESF 1438	ON493974
Cyberlindnera xylosilytica	NRRL YB-2097	EF550324
Debaryomyces hansenii	LESF 1405	ON493924

Debaryomyces hansenii	NRRL Y-7426	U45808
Debaryomyces prosopidis	JCM 9913	AB054993
Diutina catenulata	LESF 1446	ON493934
Diutina catenulata	CBS 565	KT336721
Grigorovia humatica	IFO 10673	AB040999
Grigorovia sp.	LESF 1345	ON493914
Grigorovia yakushimaensis	IFO 1889	AB041000
Hanseniaspora lachancei	CBS 8818	KY107804
Hanseniaspora opuntiae	LESF 1470	ON493938
Hanseniaspora opuntiae	CBS 8733	AJ512453
Hanseniaspora thailandica	ST-464	DQ404527
Hanseniaspora thailandica	LESF 1297	ON493932
Hyphopichia burtonii	LESF 1503	ON493977
Hyphopichia burtonii	CBS 2352	KY107882
Hyphopichia fennica	CBS 6027	KY106445
Hyphopichia heimii	NRRL Y-7502	JQ689033
Hyphopichia lachancei	CBS 5999	MN645470
Hyphopichia pseudorhagii	NRRL YB-2076	AY789656
Hyphopichia rhagii	CBS 618	KY106723
Hyphopichia sp.	LESF 1344	ON493978
Issatchenkia orientalis	NRRL Y-5396	U76347
Jamesozyma spencerorum	LESF 1468	ON493979
Jamesozyma spencerorum	NRRL Y-17920	AY048162
Kluyveromyces hubeiensis	AS 2.1536	AH013031
Kodamaea ohmeri	LESF 1474	ON493980
Kodamaea ohmeri	NRRL Y-1932	U45702

Kurtzmaniella natalensis	NRRL Y-17680	U45818
Kurtzmaniella quercitrusa	LESF 1421	ON493967
Kurtzmaniella quercitrusa	NRRL Y-5392	U45831
Kurtzmaniella sp	CLIB 1620	LN870347
Kurtzmaniella sp	LESF 1473	ON493966
Kurtzmaniella sp	LESF 1491	ON493996
Limtongozyma cylindracea	LESF 1500	ON493917
Limtongozyma cylindracea	NRRL Y-17506	EU011644
Limtongozyma sp.	LESF 1404	ON493920
Lodderomyces elongisporus	LESF 1360	ON493922
Lodderomyces elongisporus	NRRL YB-4239	LEU45763
Metschnikowia cibodasensis	UICC Y-340	AB236915
Metschnikowia koreensis	LESF 1422	ON493982
Metschnikowia koreensis	KCTC 7828	AF257272
Metschnikowia kunwiensis	CBS 9679	AJ716106
Metschnikowia reukaufii	NRRL Y-7112	U44825
Metschnikowia sp.	LESF 1355	ON493911
Metschnikowia sp.	LESF 1343	ON493912
Metschnikowia sp.	LESF 1383	ON493983
Meyerozyma caribbica	LESF 1452	ON493927
Meyerozyma caribbica	NRRL Y-27274	EU348786
Meyerozyma carpophila	LESF 1389	ON493918
Meyerozyma carpophila	CBS 5256	KY106386
Meyerozyma guilliermondii	LESF 1444	ON493925
Meyerozyma guilliermondii	NRRL Y-2075	JQ689047
Meyerozyma smithsonii	BG02-7-13-007B-1-2	AY518525

Pichia cecembensis	NRRL Y-27985	AM159112
Pichia kudriavzevii	LESF 1411	ON493941
Priceomyces fermenticarens	NRRL Y-17321	U45756
Priceomyces melissophilus	NRRL Y-7585	U45740
Priceomyces sp.	LESF 1391	ON493954
Saccharomycetales sp.	LESF 1445	ON493907
Scheffersomyces cryptocerci	NRRL Y-48824	KC702810
Scheffersomyces lignosus	ATCC 58779	HQ652022
Scheffersomyces shehatae	NRRL Y-12858	JQ025409
Scheffersomyces sp.	LESF 1457	ON493988
Schizosaccharomyces pombe	NRRL Y-12796	JQ689077
Schwanniomyces vanrijiae	LESF 1478	ON493989
Schwanniomyces vanrijiae	NRRL Y-7430	U45842
Starmerella apicola	NRRL Y-2481	U45703
Starmerella bombicola	LESF 1472	ON493929
Starmerella bombicola	NRRL Y-17069	U45705
Starmerella etchellsii	LESF 1300	ON493946
Starmerella etchellsii	NRRL Y-17084	U45723
Starmerella floris	UWO(PS)00-226.2	AF313353
Starmerella ilheusensis	UFMG-CM-Y596	KR232374
Starmerella meliponinorum	LESF 1496	ON493947
Starmerella meliponinorum	UWO(PS)00-227.1	AF313354
Starmerella sp.	LESF 1293	ON493951
Starmerella sp.	LESF 1454	ON493995
Sugiyamaella americana	NRRL YB-2067	DQ438193
Sugiyamaella bonitensis	LESF 1465	ON493990

Sugiyamaella bonitensis	UFMG-CM-Y608	KT006004
Sugiyamaella boreocaroliniensis	LESF 1366	ON493916
Sugiyamaella boreocaroliniensis	NRRL YB-1835	DQ438221
Sugiyamaella carassensis	LESF 1463	ON493991
Sugiyamaella carassensis	UFMG-CM-Y606	KX550111
Sugiyamaella floridensis	NRRL YB-3827	DQ438222
Sugiyamaella ligni	LESF 1459	ON493992
Sugiyamaella ligni	UFMG-CM-Y295	KX550112
Sugiyamaella smithiae	LESF 1336	ON493915
Sugiyamaella smithiae	NRRL Y-17850	DQ438218
Wickerhamiella azyma	CBS 6826	EF536346
Wickerhamiella occidentalis	LESF 1296	ON493930
Wickerhamiella occidentalis	CBS 8452	AF046037
Wickerhamiella sp.	LESF 1495	ON493913
Wickerhamiella sp.	LESF 1506	ON493952
Wickerhamomyces canadensis	NRRL Y-1888	EF550300
Wickerhamomyces ciferrii	LESF 1397	ON493994
Wickerhamomyces ciferrii	NRRL Y-1031	EF550339
Wickerhamomyces ochangensis	N7a-Y2T	HM485464
W. psychrolipolyticus	YS-2018 202-2	LC333101
Wickerhamomyces siamensis	DMKU-RK359	AB714248
Wickerhamomyces sp.	LESF 1460	ON493968
Yamadazyma laniorum	yHMH7	KY588136
Yamadazyma paraaseri	NYNU 1811114	MK682805
Yamadazyma paraaseri	LESF 1412	ON493961
Yamadazyma sp.	LESF 1490	ON493971

Yamadazyma tenuis	NRRL Y-1498	U45774
Yamadazyma tumulicola	T6517-9-5	AB365463
Yarrowia lipolytica	LESF 1385	ON493926
Yarrowia lipolytica	CBS 6124	AM268450
Yarrowia lipolytica	NRRL Y-2317	U45793
Yarrowia yakushimensis	CBS 10254	AM268474

Table S3. The effect of isolation source on killer yeasts activity.

**Statistics of interactions with 69 lawn strains**	
Total of interactions	12,420
**Statistics of interactions with 50 susceptible strains**	
Total of interactions	9,000
Killed phenotypes (same source)	6
Killed phenotypes (different source)	301
Not killed phenotypes (same source)	641
Not killed phenotypes (different source)	8,052

Pearson's Chi-squared test, $\chi 2 = 13.05$, df = 1, p-value = $3.03E^{-04}$.

Table S4. The effect of pH on the growth inhibition of *Kodamaea ohmeri* LESF 1474 and *Candida castyelli* by *Candida sinolaborantium* LESF 1467.

	Kodamaea ohmeri	*Candida castelli*
Treatment	Area in cm² (Mean ± SE)	
pH 3.5	1.03 ± 0.04	6.65 ± 0.33
pH 4.0	1.18 ± 0.04	8.61 ± 0.65
pH 4.5	2.35 ± 0.06	8.15 ± 0.92
pH 5.0	3.25 ± 0.09	5.74 ± 1.58
pH 5.5	0.81 ± 0.03	2.08 ± 0.38
pH 6.0	0	1.90 ± 0.16
pH 6.5	0	1.57 ± 0.11

Table S5. The effect of temperature on the growth inhibition of *Kodamaea ohmeri* LESF 1474 and *Candida castyelli* by *Candida sinolaborantium* LESF 1467.

	Kodamaea ohmeri	*Candida castelli*
Treatment	Area in cm² (Mean ± SE)	
17°C	2.03 ± 0.04	8.15 ± 0.73
20°C	2.42 ± 0.08	8.42 ± 0.47
25°C	2.15 ± 0.05	8.02 ± 0.49
30°C	1.01 ± 0.03	5.39 ± 0.10

Table S6. The ratio between elongated and normal *Kodamaea ohmeri* LESF 1474 cells challenged with killer toxins.

Treatment	Value (Mean ± SE)
Control	0.03 ± 0.00
LESF1467	0.24 ± 0.02
Klus	0.49 ± 0.07

LESF1467 × Control: Welch Two Sample t-test, t = -12.18, df = 9.26, p-value = $5.194E^{-07}$
Klus × Control: Welch Two Sample t-test, t = -6.78, df = 9.02, p-value = $8.034E^{-05}$

Table S7. Cell survival of *Kodamaea ohmeri* LESF 1474 cells against killer toxins.

Treatment	CFUs (Mean ± SE)
Control	$1.47E^{+02} \pm 1.31E^{+01}$
LESF1467	$7.00E^{+01} \pm 6.33E^{+00}$
Klus	$1.30E^{+02} \pm 1.02E^{+01}$

LESF1467 × Control: Welch Two Sample t-test, t = 5.30, df = 12.99, p-value = $1.45E^{-04}$
Klus × Control: Two Sample t-test, t = 1.02, df = 18, p-value = 0.3194

Table S8. Statistics of the assembly, prediction, and annotation of the *Candida sinolaborantium* LESF1467 genome.

**Statistics of cleaning steps**		
Category	Before filtering	After filtering
Total of reads	5,240,040	4,203,746
Total of bases	786,006,000	608,646,389
Ratio Q30	69.15%	75.61%
GC contente	46.15%	44.46%
**BUSCO and QUAST assembly statistics**		
Category	Value	
Total BUSCO groups searched (saccharomycetes_odb10)	2,137	
Complete BUSCOs	2,097 (98.2%)	
Complete and simple copy BUSCOs	2,099 (98.1%)	
Complete and duplicates BUSCOs	2 (0.1%)	
Fragmented BUSCOs	1.00%	
Absent BUSCOs	0.80%	
Contigs > 200 bp	270	
Contigs > 1,000 bp	163	
Total length (>= 0 bp)	11,214,429	
Total length (>= 1,000 bp)	11,175,526	
Largest contig	543,051	
GC (%)	48.76	
N50	155,979	
N's per 100 kbp	29.07	
**Gene prediction and annotation statistics**		
Category	Value	
Number of protein-coding genes	5,951	

Table S9. Killer toxins used as queries for the BLASTp search on *Candida sinolaborantium* LESF1467 genome.

Toxin	Organism	GenBank accession
HM-1	*Cyberlindnera mrakii*	P10410
SMK	*Millerozyma farinosa*	P19972
K1	*Saccharomyces cerevisiae*	AII19505
K2	*Saccharomyces cerevisiae*	CAA39941
KHR	*Saccharomyces cerevisiae*	P22313
KHS	*Saccharomyces cerevisiae*	P39690
Klus	*Saccharomyces cerevisiae*	ADG64740
K1L	*Saccharomyces paradoxus*	QQX23408
K21	*Saccharomyces paradoxus*	ATN38270
K28	*Saccharomyces paradoxus*	ATN38273
K28	*Saccharomyces paradoxus*	AII19507
K45	*Saccharomyces paradoxus*	ATN38271
K62	*Saccharomyces paradoxus*	ATN38496
K66	*Saccharomyces paradoxus*	AYN80721
K74	*Saccharomyces paradoxus*	ATN38272
Kbarr-1	*Torulaspora delbrueckii*	ALI93476
KP4	*Ustilago maydis*	Q90121
zygocin	*Zygosaccharomyces bailii*	AAM54023

Table S10. Blastp hits of killer toxins towards *Candida sinolaborantium* LESF1467 genome.

Query	% Identity	Alignment length	Mismatches	e-value	Bit score
K2 (CAA39941)	25.15	167	118	3.16E-07	49.3
K21 (ATN38270)	27.35	117	60	1.40E+00	28.5
K21 (ATN38270)	24.62	130	90	5.20E+00	26.9
K28 (AII19507)	23.59	106	69	7.60E+00	26.2
K28 (ATN38273)	24.38	160	99	4.30E+00	27.3
K45 (ATN38271)	28.21	117	57	9.30E+00	26.2
K66 (AYN80721)	26.52	132	85	8.70E+00	26.2
K74 (ATN38272)	25.52	145	76	9.10E+00	26.2
KHR (P22313)	23.53	187	101	7.30E+00	26.2
KHS (P39690)	26.98	126	78	3.50E+00	28.9
KHS (P39690)	24.00	100	75	5.90E+00	28.1
Klus (ADG64740)	27.82	151	94	1.56E-05	42.4

Table S11. Statistics of Blastp hits of Ksino homologs.

GenBank accession	% Identity	Alignment length	e-value	Species	Family	Class	Phylum
XP_022390945	33.33	144	2.34E-08	*Aspergillus bombycis*	Aspergillaceae	Eurotiomycetes	Ascomycota
XP_031921194	35.17	145	2.88E-09	*Aspergillus caelatus*	Aspergillaceae	Eurotiomycetes	Ascomycota
KAB8067042	31.97	147	1.30E-08	*Aspergillus leporis*	Aspergillaceae	Eurotiomycetes	Ascomycota
KAE8410747	35.17	145	1.86E-09	*Aspergillus pseudocaelatus*	Aspergillaceae	Eurotiomycetes	Ascomycota
XP_031911149	40.00	110	1.61E-10	*Aspergillus pseudotamarii*	Aspergillaceae	Eurotiomycetes	Ascomycota
XP_041552517	33.03	109	7.00E-03	*Aspergillus puulaauensis*	Aspergillaceae	Eurotiomycetes	Ascomycota
KAE8167611	32.65	147	1.26E-08	*Aspergillus tamarii*	Aspergillaceae	Eurotiomycetes	Ascomycota
TEY44878	36.49	148	1.88E-10	*Botryotinia calthae*	Sclerotiniaceae	Leotiomycetes	Ascomycota
KAF7887508	37.01	154	8.86E-12	*Botryotinia globosa*	Sclerotiniaceae	Leotiomycetes	Ascomycota

KAF7906842	35.14	148	1.94E-10	*Botryotinia globosa*	Sclerotiniaceae	Leotiomycetes	Ascomycota
KAF7958093	33.56	149	1.61E-08	*Botrytis aclada*	Sclerotiniaceae	Leotiomycetes	Ascomycota
XP_038810106	34.87	152	3.70E-10	*Botrytis deweyae*	Sclerotiniaceae	Leotiomycetes	Ascomycota
XP_037198077	32.20	118	1.00E-03	*Botrytis fragariae*	Sclerotiniaceae	Leotiomycetes	Ascomycota
CAK7894508	37.24	196	2.03E-34	*Candida anglica*	Debaryomycetaceae	Pichiomycetes	Ascomycota
CAK7918227	37.24	196	8.83E-35	*Candida anglica*	Debaryomycetaceae	Pichiomycetes	Ascomycota
XP_044657970	35.95	153	2.04E-10	*Cercospora kikuchii*	Mycosphaerellaceae	Dothideomycetes	Ascomycota
TDZ15916	32.65	147	4.82E-07	*Colletotrichum orbiculare*	Mycosphaerellaceae	Dothideomycetes	Ascomycota
TDZ36561	32.65	147	2.88E-07	*Colletotrichum spinosum*	Mycosphaerellaceae	Dothideomycetes	Ascomycota
TDZ71680	33.33	147	4.78E-08	*Colletotrichum trifolii*	Mycosphaerellaceae	Dothideomycetes	Ascomycota
XP_047769465	31.91	141	9.70E-09	*Fulvia fulva*	Mycosphaerellaceae	Dothideomycetes	Ascomycota
KAG5657935	36.09	133	2.72E-11	*Fusarium avenaceum*	Nectriaceae	Sordariomycetes	Ascomycota
KAF5988856	32.99	194	1.76E-09	*Fusarium coicis*	Nectriaceae	Sordariomycetes	Ascomycota
PTD03240	30.77	156	9.14E-10	*Fusarium culmorum*	Nectriaceae	Sordariomycetes	Ascomycota
KAF4961169	30.38	158	6.92E-08	*Fusarium gaditjirri*	Nectriaceae	Sordariomycetes	Ascomycota
CZS79277	31.41	156	9.22E-10	*Fusarium graminearum*	Nectriaceae	Sordariomycetes	Ascomycota
KAK6720909	31.41	156	8.76E-10	*Fusarium graminearum*	Nectriaceae	Sordariomycetes	Ascomycota
WXC44248	31.41	156	7.59E-10	*Fusarium graminearum*	Nectriaceae	Sordariomycetes	Ascomycota
XP_011319547	31.41	156	7.20E-10	*Fusarium graminearum*	Nectriaceae	Sordariomycetes	Ascomycota
CAF3522722	31.17	154	1.15E-08	*Fusarium graminearum*	Nectriaceae	Sordariomycetes	Ascomycota
KAI6763338	31.17	154	1.09E-08	*Fusarium graminearum*	Nectriaceae	Sordariomycetes	Ascomycota
KAJ4009560	31.41	156	7.84E-10	*Fusarium irregulare*	Nectriaceae	Sordariomycetes	Ascomycota
RGP71720	32.05	156	1.64E-10	*Fusarium longipes*	Nectriaceae	Sordariomycetes	Ascomycota
RGP78867	35.95	153	1.11E-11	*Fusarium longipes*	Nectriaceae	Sordariomycetes	Ascomycota
KAF5724847	30.06	163	2.08E-06	*Fusarium mundagurra*	Nectriaceae	Sordariomycetes	Ascomycota
XP_044675207	35.50	169	2.36E-10	*Fusarium musae*	Nectriaceae	Sordariomycetes	Ascomycota
KAF5567016	33.73	169	1.03E-10	*Fusarium napiforme*	Nectriaceae	Sordariomycetes	Ascomycota

PNP74207	31.01	158	3.92E-08	*Fusarium nygamai*	Nectriaceae	Sordariomycetes	Ascomycota
KAG7405468	31.79	195	8.58E-09	*Fusarium oxysporum*	Nectriaceae	Sordariomycetes	Ascomycota
EXM19552	31.48	162	6.45E-09	*Fusarium oxysporum*	Nectriaceae	Sordariomycetes	Ascomycota
KAF5558678	30.43	161	1.05E-04	*Fusarium phyllophilum*	Nectriaceae	Sordariomycetes	Ascomycota
KAF5609400	31.01	158	5.77E-08	*F. pseudoanthophilum*	Nectriaceae	Sordariomycetes	Ascomycota
KAF5592859	35.50	169	1.80E-09	*Fusarium pseudocircinatum*	Nectriaceae	Sordariomycetes	Ascomycota
QPC77963	32.05	156	2.98E-10	*F. pseudograminearum*	Nectriaceae	Sordariomycetes	Ascomycota
UZP36555	31.41	156	9.71E-10	*F. pseudograminearum*	Nectriaceae	Sordariomycetes	Ascomycota
XP_009254827	31.41	156	7.67E-10	*F. pseudograminearum*	Nectriaceae	Sordariomycetes	Ascomycota
XP_037208290	33.14	169	7.70E-10	*Fusarium tjaetaba*	Nectriaceae	Sordariomycetes	Ascomycota
KAH7251039	33.33	147	3.24E-08	*Fusarium tricinctum*	Nectriaceae	Sordariomycetes	Ascomycota
KAG5800341	31.79	195	1.28E-08	*Fusarium xylarioides*	Nectriaceae	Sordariomycetes	Ascomycota
KAG5820874	32.99	194	6.82E-09	*Fusarium xylarioides*	Nectriaceae	Sordariomycetes	Ascomycota
KAH7009605	33.78	148	5.84E-11	*Ilyonectria destructans*	Nectriaceae	Sordariomycetes	Ascomycota
XP_046102592	34.46	148	1.34E-12	*Ilyonectria robusta*	Nectriaceae	Sordariomycetes	Ascomycota
KAH6972811	36.04	111	5.15E-10	*Ilyonectria* sp.	Nectriaceae	Sordariomycetes	Ascomycota
XP_003958467	39.90	198	1.70E-31	*Kazachstania africana*	Saccharomycetaceae	Saccharomycetes	Ascomycota
XP_003957740	36.78	174	4.53E-27	*Kazachstania africana*	Saccharomycetaceae	Saccharomycetes	Ascomycota
XP_003957274	33.06	124	1.12E-04	*Kazachstania africana*	Saccharomycetaceae	Saccharomycetes	Ascomycota
KAJ8128189	31.90	116	6.00E-03	*Lasiodiplodia mahajangana*	Botryosphaeriaceae	Dothideomycetes	Ascomycota
MCJ1271286	34.76	164	2.13E-11	*Lobaria immixta*	Peltigerineae	Lecanoromycetes	Ascomycota
XP_003671032	31.97	122	1.81E-04	*Naumovozyma dairenensis*	Saccharomycetaceae	Saccharomycetes	Ascomycota
XP_057019693	30.23	129	1.74E-04	*Penicillium verhagenii*	Aspergillaceae	Eurotiomycetes	Ascomycota
MCJ1467775	31.10	164	7.55E-08	*Pseudocyphellaria aurata*	Peltigerineae	Lecanoromycetes	Ascomycota
XP_023627751	33.94	109	4.76E-06	*Ramularia collo_cygni*	Mycosphaerellaceae	Dothideomycetes	Ascomycota
XP_001383947	42.02	188	2.48E-35	*Scheffersomyces stipitis*	Debaryomycetaceae	Pichiomycetes	Ascomycota
XP_001388021	37.06	143	7.55E-17	*Scheffersomyces stipitis*	Debaryomycetaceae	Pichiomycetes	Ascomycota

XP_001588904	35.86	145	2.58E-08	*Sclerotinia sclerotiorum*	Sclerotiniaceae	Leotiomycetes	Ascomycota
XP_020062392	35.08	191	4.32E-25	*Suhomyces tanzawaensis*	Debaryomycetaceae	Pichiomycetes	Ascomycota
KAH6887094	32.21	149	2.98E-09	*Thelonectria olida*	Nectriaceae	Sordariomycetes	Ascomycota
XP_056028500	33.56	146	1.12E-08	*Trichoderma breve*	Hypocreaceae	Sordariomycetes	Ascomycota
KAK4063601	31.61	155	2.93E-08	*Trichoderma harzianum*	Hypocreaceae	Sordariomycetes	Ascomycota
KKO99952	32.88	146	9.39E-08	*Trichoderma harzianum*	Hypocreaceae	Sordariomycetes	Ascomycota
KAF3056720	32.88	146	1.34E-08	*Trichoderma lentiforme*	Hypocreaceae	Sordariomycetes	Ascomycota
KAH0526984	33.56	146	1.18E-08	*Trichoderma semiorbis*	Hypocreaceae	Sordariomycetes	Ascomycota
KAJ7348369	31.94	144	2.32E-07	*Mycena albidolilacea*	Marasmiineae	Agaricomycetes	Basidiomycota
KAJ7036171	32.26	155	3.97E-07	*Mycena alexandri*	Marasmiineae	Agaricomycetes	Basidiomycota
KAJ7048476	30.99	171	1.25E-07	*Mycena amicta*	Marasmiineae	Agaricomycetes	Basidiomycota
KAJ7051285	31.25	144	2.43E-07	*Mycena amicta*	Marasmiineae	Agaricomycetes	Basidiomycota
KAJ6568641	32.26	124	3.00E-03	*Mycena capillaripes*	Marasmiineae	Agaricomycetes	Basidiomycota
KAJ7124264	32.64	144	4.13E-05	*Mycena epipterygia*	Marasmiineae	Agaricomycetes	Basidiomycota
KAJ7463168	30.19	159	1.87E-08	*Mycena latifolia*	Marasmiineae	Agaricomycetes	Basidiomycota
KAJ7436857	30.32	155	8.19E-06	*Mycena latifolia*	Marasmiineae	Agaricomycetes	Basidiomycota
KAJ7478936	30.32	155	3.00E-03	*Mycena latifolia*	Marasmiineae	Agaricomycetes	Basidiomycota
KAJ7453852	31.45	124	3.00E-02	*Mycena latifolia*	Marasmiineae	Agaricomycetes	Basidiomycota
KAF7336346	30.97	155	1.16E-04	*Mycena venus*	Marasmiineae	Agaricomycetes	Basidiomycota
KAJ6517861	30.28	142	2.48E-07	*Mycena vulgaris*	Marasmiineae	Agaricomycetes	Basidiomycota
KAJ7614860	32.43	148	7.44E-07	*Roridomyces roridus*	Marasmiineae	Agaricomycetes	Basidiomycota

Table S12. SWISS-MODEL structure assessment scores of Ksino top unrelaxed model and Klus top unrelaxed model compared to models after 100 ns of molecular dynamics simulation. Global pLDDT score is also included in the last row.

	**Ksino unrelaxed**	**Ksino relaxed**	**Ksino 100ns**	**Klus unrelaxed**	**Klus relaxed**	**Klus 100ns**
MolProbity score (version 4.4)	3.44	2.39	1.54	3.51	2.15	1.86
Clash score	32.64	5.43	1.03	37.61	2.97	1.1
Ramachandran favoured	77.00%	87.50%	91.16%	72.92%	82.08%	89.04%
Ramachandran outliers	15.50%	5.50%	0.55%	17.50%	7.08%	1.83%
Rotamer outliers	7.50%	4.38%	1.99%	6.86%	2.94%	4.19%
Bad bonds	64/1545	0/1546	28/1534	57/1936	0/1937	34/1925
Bad angles	119/2094	14/2096	77/2063	119/2651	275273	122/2619
Twisted non-proline	22/196	4/196	1/196	12/235	4/235	2/235
Global pLDDT	63645	-	-	65086	-	-

Table S13. The effect of pH on the growth inhibition of *Kodamaea ohmeri* LESF 1474 and *Candida castyelli* by Ksino expressed by *S. cerevisiae*.

	Kodamaea ohmeri	*Candida castelli*
Treatment	Area in cm² (Mean ± SE)	
pH 3.5	0	3.65 ± 0.57
pH 4.0	0	8.42 ± 0.70
pH 4.5	0	7.29 ± 1.26
pH 5.0	0	3.30 ± 0.06
pH 5.5	0	0
pH 6.0	0	0
pH 6.5	0	0

Table S14. The effect of temperature on the growth inhibition of *Kodamaea ohmeri* LESF 1474 and *Candida castyelli* by Ksino expressed by *S. cerevisiae*.

	Kodamaea ohmeri	*Candida castelli*
Treatment	Area in cm² (Mean ± SE)	
17°C	0	2.18 ± 0.56
20°C	0	7.35 ± 0.20
25°C	0	7.53 ± 0.72
30°C	0	1.75 ± 0.17
35°C	0	0

Table S15. Yeasts used in killer assays as susceptible or killer positive standards.

**Yeast species**	**Isolate**	**Use in the study**
Blastobotrys proliferans	LESF 1481	Lawn strain
Candida blattae	LESF 1417	Lawn strain
Candida railenensis	LESF 1437	Lawn strain
Candida railenensis	LESF 1551	Lawn strain
Candida sinolaborantium	LESF 1467	Lawn strain
Cyberlindnera sp.	LESF 1556	Lawn strain
Cyberlindnera sp.	LESF 1555	Lawn strain
Debaryomyces hansenii	LESF 1405	Lawn strain
Diutina catenulata	LESF 1446	Lawn strain
Hanseniaspora opuntiae	LESF 1470	Lawn strain

Hanseniaspora thailandica	LESF 1297	Lawn strain
Issatchenkia orientalis	LESF 1411	Lawn strain
Jamesozyma spencerorum	LESF 1468	Lawn strain
Kodamaea ohmeri	LESF 1474	Lawn strain
Kurtzmaniella quercitrusa	LESF 1489	Lawn strain
Limtongozyma cylindracea	LESF 1500	Lawn strain
Metschnikowia koreensis	LESF 1422	Lawn strain
Meyerozyma caribbica	LESF 1452	Lawn strain
Meyerozyma caribbica	LESF 1545	Lawn strain
Meyerozyma carpophila	LESF 1389	Lawn strain
Meyerozyma guilliermondii	LESF 1406	Lawn strain
Ogataea sp.	LESF 1525	Lawn strain
Schwanniomyces vanrijiae	LESF 1521	Lawn strain
Starmerella bombicola	LESF 1472	Lawn strain
Starmerella etchellsii	LESF 1300	Lawn strain
Starmerella meliponinorum	LESF 1496	Lawn strain
Sugiyamaella bonitensis	LESF 1465	Lawn strain
Sugiyamaella carassensis	LESF 1463	Lawn strain
Sugiyamaella ligni	LESF 1459	Lawn strain
Wickerhamomyces ciferrii	LESF 1397	Lawn strain
Yamadazyma paraaseri	LESF 1412	Lawn strain
Yamadazyma riverae	LESF 1541	Lawn strain
Yamadazyma riverae	LESF 1538	Lawn strain
Yarrowia lipolytica	LESF 1385	Lawn strain
Candida castellii	Y-17070	Lawn strain
Saccharomyces arboricolus	2.3317	Lawn strain
Kazachstania africana	Y-2729	Lawn strain

Saccharomyces cerevisiae	Y-2046	Lawn strain
Saccharomyces cerevisiae	MSY"99"	Lawn strain
Saccharomyces cerevisiae	Y-5509	Lawn strain
Saccharomyces cerevisiae	Y-1891	Lawn strain
Saccharomyces cerevisiae	BY 4741	Lawn strain
Saccharomyces mikatae	NBRC 1815	Lawn strain
Saccharomyces paradoxus	YB - 4565	Lawn strain
Saccharomyces paradoxus	Y8.5	Lawn strain
Saccharomyces eubayanus	CBS 12357	Lawn strain
Saccharomyces kudriavzevii	NBRC/IFO 10990	Lawn strain
Naumovozyma castellii	NCYC2898	Lawn strain
Nakaseomyces delphensis	Y-2379	Lawn strain
Saccharomyces cerevisiae	YJM1307	K1 toxin-producing strain
Saccharomyces cerevisiae	CYC1172	K2 toxin-producing strain
Saccharomyces cerevisiae	DSM-70459	Klus toxin-producing strain
Saccharomyces cerevisiae	OS169	K62 toxin-producing strain
Saccharomyces cerevisiae	OS78	K45 toxin-producing strain
Saccharomyces cerevisiae	OS294	K74 toxin-producing strain
Saccharomyces cerevisiae	MSC300c	K28 toxin-producing strain
Saccharomyces cerevisiae	OS40	K21 toxin-producing strain
Saccharomyces paradoxus	Y-63717	K1L toxin-producing strain
Candida albicans	Y-12983	Pathogenic lawn strain
Candida auris	AR0381	Pathogenic lawn strain
Candida auris	AR0382	Pathogenic lawn strain
Candida auris	AR0383	Pathogenic lawn strain
Candida auris	AR0384	Pathogenic lawn strain
Candida auris	AR0385	Pathogenic lawn strain

Candida auris	AR0386	Pathogenic lawn strain
Candida auris	AR0387	Pathogenic lawn strain
Candida auris	AR0388	Pathogenic lawn strain
Candida auris	AR0389	Pathogenic lawn strain
Candida auris	AR0390	Pathogenic lawn strain
Candida castellii	Y-17070	Pathogenic lawn strain
Candida glabrata	AR0323	Pathogenic lawn strain
Candida glabrata	AR0317	Pathogenic lawn strain
Candida glabrata	ATCC2001	Pathogenic lawn strain
Candida humilis	Y-17074	Pathogenic lawn strain
Candida kefyr	AR0588	Pathogenic lawn strain
Candida nivarensis	Y-48269	Pathogenic lawn strain
Candida pararugose	AR0587	Pathogenic lawn strain
Saccharomyces cerevisiae	AR0400	Pathogenic lawn strain
